# Fast hydrogen purification through graphitic carbon nitride nanosheet membranes

**DOI:** 10.1038/s41467-022-33654-6

**Published:** 2022-10-04

**Authors:** Yisa Zhou, Ying Wu, Haoyu Wu, Jian Xue, Li Ding, Rui Wang, Haihui Wang

**Affiliations:** 1 School of Chemistry and Chemical Engineering, Guangdong Provincial Key Lab of Green Chemical Product Technology, South China University of Technology, Guangzhou, 510640 China; 2 Beijing Key Laboratory of Membrane Materials and Engineering, Department of Chemical Engineering, Tsinghua University, Beijing, 100084 China

**Keywords:** Chemical engineering, Two-dimensional materials, Two-dimensional materials, Structural properties

## Abstract

Two-dimensional graphitic carbon nitride (g-C_3_N_4_) nanosheets are ideal candidates for membranes because of their intrinsic in-plane nanopores. However, non-selective defects formed by traditional top-down preparation and the unfavorable re-stacking hinder the application of these nanosheets in gas separation. Herein, we report lamellar g-C_3_N_4_ nanosheets as gas separation membranes with a disordered layer-stacking structure based on high quality g-C_3_N_4_ nanosheets through bottom-up synthesis. Thanks to fast and highly selective transport through the high-density sieving channels and the interlayer paths, the membranes, superior to state-of-the-art ones, exhibit high H_2_ permeance of 1.3 × 10^−6 ^mol m^−2^ s^−1^ Pa^−1^ with excellent selectivity for multiple gas mixtures. Notably, these membranes show excellent stability under harsh practice-relevant environments, such as temperature swings, wet atmosphere and long-term operation of more than 200 days. Therefore, such lamellar membranes with high quality g-C_3_N_4_ nanosheets hold great promise for gas separation applications.

## Abstract (web summary)

In this work, lamellar graphitic carbon nitride nanosheet membranes are constructed for gas separation. Benefiting from their high-density intrinsic in-plane nanopores and broader permeable interlayer channels, the proposed membranes exhibit high H_2_ permeance with good selectivity of multiple gas mixtures.

## Introduction

Carbon dioxide (CO_2_) concentrations in the atmosphere have rapidly increased in the last decades due to the consumption of fossil fuels, which have caused various global climate issues^1–3^. Therefore, the Paris Agreement was reached to stipulate that carbon neutrality should be achieved in the second half of the 21st century^4^. As a carbon-free energy carrier, hydrogen is now widely considered the next generation energy to reduce CO_2_ emissions^5^. However, most produced hydrogen is mixed with larger molecules like CO_2_, N_2_, CH_4_, which needs further purification before practical applications^6^. Nowadays, traditional industrial separation methods such as distillation and pressure swing adsorption are energy-intensive. As an emerging technology, membranes separation offers an efficient energy-saving alternative for H_2_ purification^7,8^. However, the development of traditional polymer membranes is limited by the trade-off relationship between permeability and selectivity (known as Robeson’s upper bounds)^9^. Recently, two-dimensional (2D) nanosheets have offered a superb building platform for membrane construction owing to their nanometer-thin thickness^10–13^. Graphitic carbon nitride (g-C_3_N_4_) nanosheets, hosting high-density molecular-sized pores composed of tri-s-triazine units in the entire 2D plane^14,15^, are considered ideal building blocks for molecular sieving membrane. Benefits to the nanometer-thin thickness and high-density nanopores, g-C_3_N_4_ nanosheet membranes should be a superior candidate for H_2_ separation^16,17^, which was already proved by theoretical investigations. For instance, Li et al.^18^ found that H_2_ had the lowest diffusion barrier to go through the g-C_3_N_4_ nanosheets by density functional theory (DFT) calculations, which resulted in a superior selectivity between H_2_ and other larger gases. Moreover, Guo et al.^19^ predicted a high H_2_ permeance (13 mol m^−2^ s^−1^ Pa^−1^) across a bilayer g-C_3_N_4_ nanosheets membrane by molecular dynamics (MD) simulation. Therefore, the prospect of developing g-C_3_N_4_ nanosheets membrane is highly attractive.

Nevertheless, only a few g-C_3_N_4_ nanosheet membranes have been reported for gas separation. One reason for this is the difficulty of obtaining high-quality g-C_3_N_4_ nanosheets. Similar to other 2D materials, most g-C_3_N_4_ nanosheets are usually exfoliated from bulk materials by top-down methods^20,21^. During the top-down exfoliation processes, harsh environments (oxidative atmosphere or acid solution) were applied to destroy the interlayer interactions in bulk g-C_3_N_4_, and thereby obtaining single or few-layer nanosheets^22,23^. However, the structural deterioration may occur simultaneously during this exfoliation process, leading to the formation of larger defects in g-C_3_N_4_ nanosheets. For example, Wang et al.^24^ fabricated g-C_3_N_4_ nanosheet membranes containing 1.5–3 nm artificial nanopores through the top-down method using concentrated hydrochloric acid. These membranes exhibited excellent performance in nanofiltration, while the artificial nanopores were too large for H_2_ purification. On the other hand, it was reported that g-C_3_N_4_ nanosheets tended to re-stack to form tight films due to strong π–π interaction^25^, leading to the blockage of intrinsic in-plane nanopores. By adding polybenzimidazole chains as spaces in g-C_3_N_4_ nanosheets to prevent the re-stacking, Villalobos et al.^26^ developed a g-C_3_N_4_ nanosheets-based mixed matrix membrane with considerable separation performance. However, the merits of g-C_3_N_4_ nanosheets can not be exploited in the mixed matrix membranes.

Hence, in this work, high-quality g-C_3_N_4_ nanosheets are prepared by a bottom-up method where the produced g-C_3_N_4_ has been delaminated into nanosheets along with the thermal polycondensation, skipping the exfoliation step, thus significantly avoiding the structural deterioration of g-C_3_N_4_ nanosheets. Then the isopropanol is used as a dispersant for membrane preparation to weaken the π–π interaction between g-C_3_N_4_ nanosheets. As a result, laminar g-C_3_N_4_ membranes with disordered stacking structures assembled by high-quality g-C_3_N_4_ nanosheets are obtained. The lamellar g-C_3_N_4_ nanosheet membranes exhibit excellent H_2_ permeance of 1.3 × 10^−6^ mol m^−2^ s^−1^ Pa^−1^ with high selectivity in mixed-gas separation. Furthermore, the gas separation mechanism through the g-C_3_N_4_ membrane is revealed by DFT and MD simulations, which is based on the synergistic influence of size exclusion and the interactions between gas molecules and g-C_3_N_4_ nanosheets. Therefore, this work not only prepares a lamellar g-C_3_N_4_ nanosheets membrane for efficiently H_2_ purification but also provides a strategy for constructing other 2D nanosheets membranes for gas separation.

## Results

### Fabrication of high-quality g-C_3_N_4_ nanosheets

**Fig. 1:**
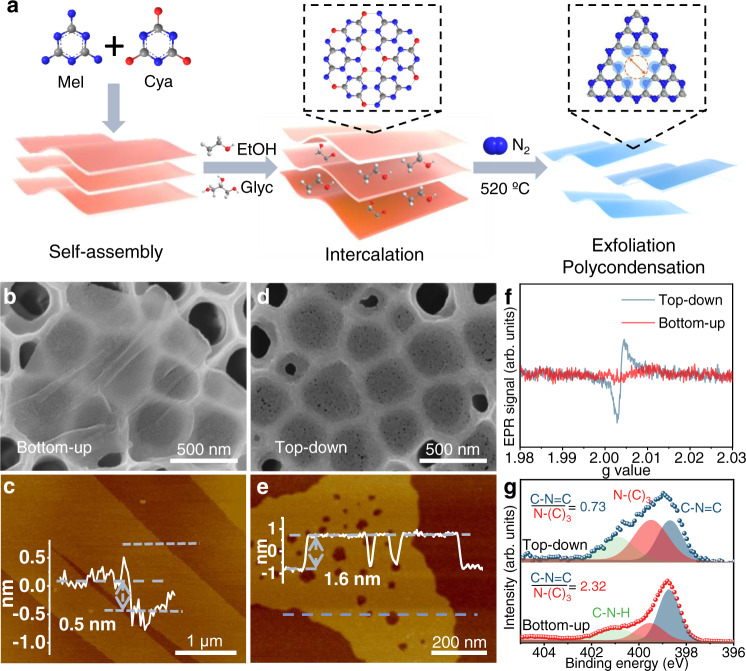
High-quality g-C_3_N_4_ nanosheets. **a** The fabrication process of g-C_3_N_4_ nanosheets. Step 1: The melamine (Mel) and cyanuric acid (Cya) were self-assembled into layered supramolecular precursors under hydrothermal conditions. Step 2: Through intercalation of an ethanol (EtOH)/glycerol (Glyc) mixture (3:1), the layers were expanded. Step 3: The g-C_3_N_4_ nanosheets were obtained via a calcination process under nitrogen. C, N, O, and H atoms are colored in gray, blue, red, and white, respectively. Morphology of bottom-up g-C_3_N_4_ nanosheets: **b** SEM image and **c** AFM image with relative height profile. Morphology of top-down g-C_3_N_4_ nanosheets: **d** SEM image and **e** AFM image with relative height profile. **f** Room-temperature EPR spectra and **g** N 1*s* XPS spectra of bottom-up and top-down g-C_3_N_4_ nanosheets. Source data are provided as a Source data file.

The detailed steps of the bottom-up method are shown in Fig. 1a. First, melamine (Mel) and cyanuric acid (Cya) are self-assembled into layered supramolecular precursors (Supplementary Fig. 1). Then, the ethanol (EtOH)/glycerol (Glyc) mixture are intercalated into the layered precursors. During subsequent calcination in an inert gas, the g-C_3_N_4_ is formed through thermal polycondensation. At the same time, the released gases (such as EtOH, Glyc, NO_x_, and H_2_O) produced by evaporation and decomposition of the intercalated molecules lead to the exfoliation of the layered precursors. Different from the top-down method (Supplementary Fig. 2), no further exfoliation is required, hence the structural deterioration of the g-C_3_N_4_ nanosheets can be greatly avoided. The obtained g-C_3_N_4_ nanosheets by the bottom-up method were first examined using ^13^C solid-state nuclear magnetic resonance (NMR). The spectrum of the g-C_3_N_4_ nanosheets exhibits two signal groups at 163 and 155 ppm, corresponding to the CN_2_(NH_x_) carbon atoms (C1) and CN_3_ carbon atoms (C2), respectively^27^, indicating the successful synthesis of g-C_3_N_4_ (Supplementary Fig. 3). The Fourier transform infrared spectroscopy (FTIR) of both types of nanosheets (Supplementary Fig. 4a) shows the typical molecular structure of g-C_3_N_4_^14^. The signal located at 810 cm^−1^ is originated from the characteristic tri-s-triazine breathing mode. The wide bands at 1690–1150 cm^−1^ and 3680–2970 cm^−1^ can be assigned to the stretching vibrations of C–N heterocycles and the N–H stretching vibrations of the terminal -NH_2_/NH of g-C_3_N_4_, respectively. In the X-ray diffraction (XRD) patterns, the (100) peak at 12.9° stems from the lattice planes along c-axis due to the 2D planer disorder^28^ (Supplementary Figs. 4b and 5). It was reported that the disordered g-C_3_N_4_ structure was formed by the incompletely condensed tri-s-triazine units connected by hydrogen bonds through -NH_2_/NH at their edges^29^. However, this is not detected in the g-C_3_N_4_ nanosheets prepared using the top-down method, indicating that the in-planar atomic structure is destroyed. The differences between the two nanosheets were confirmed by scanning electron microscopy (SEM) and atomic force microscopy (AFM) images. As shown in Fig. 1b, c, g-C_3_N_4_ nanosheets with a thickness of approximately 0.5 nm are almost transparent to electron beams. In the case of the 0.326 nm g-C_3_N_4_ single layer^30^, the obtained g-C_3_N_4_ nanosheet with a thickness less than double layers can be regarded as a single-layer nanostructure. The increased thickness is likely due to a “dead layer” caused by the adsorbed water between the sample and the substrate or the presence of surface adsorbates such as water molecules^31^. More importantly, no apparent nanopore defects are observed in the g-C_3_N_4_ nanosheets fabricated via this bottom-up technology (Fig. 1b, c and Supplementary Fig. 6), which is desirable for molecular sieving of gases. In contrast, large defects are inevitable in nanosheets prepared by traditional top-down thermal oxidation processes due to structural degradation. As shown in Fig. 1d, e, the g-C_3_N_4_ nanosheets obtained by the classical top-down technique contain distinct randomly distributed artificial defect pores (10–100 nm).

Furthermore, the Electron paramagnetic resonance (EPR), X-ray photoelectron spectroscopy (XPS), and element analysis (EA) of the two types of g-C_3_N_4_ nanosheets were investigated to identify defect concentrations in nanosheets. As presented in Fig. 1f, a lower EPR peak intensity in bottom-up nanosheets is observed, which can be assignable to unpaired electrons, indicating fewer defects in bottom-up g-C_3_N_4_ nanosheets^32^. The N 1*s* XPS spectra (Fig. 1g) shows three peaks at 398.7, 399.6, and 400.9 eV, which can be attributed to C–N = C, N–(C)_3_, and C–NH, respectively^33^. The C–N=C to N–(C)_3_ ratio drastically decreases from 2.32 (bottom-up method) to 0.73 (top-down approach), which also suggests a lower defect concentration in the bottom-up g-C_3_N_4_ nanosheets^34^. In the EA results, the C/N atom ratio decreases from 0.68 (bottom-up method) to 0.65 (top-down approach), further confirming fewer defects in the bottom-up nanosheets (Supplementary Table 1)^32^. In addition, all g-C_3_N_4_ nanosheets obtained via the bottom-up and top-down methods are homogenously dispersed with apparent Tyndall effects (Supplementary Fig. 7). The g-C_3_N_4_ nanosheet suspensions show a clear linear relationship between UV/Vis spectroscopic absorbance and nanosheet concentration, which can be applied to determine the nanosheet concentration for membrane fabrication (Supplementary Fig. 8).

### Fabrication of lamellar g-C_3_N_4_ membranes

**Fig. 2:**
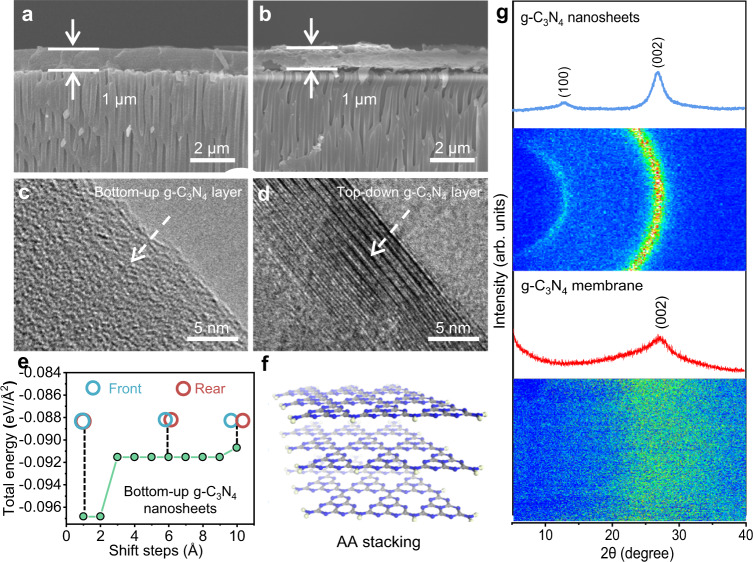
g-C_3_N_4_ nanosheets membranes with disordered layer-stacking structure. Cross-sectional SEM images of the **a** bottom-up g-C_3_N_4_ and **b** top-down g-C_3_N_4_ membranes. Cross-sectional TEM images of the **c** bottom-up g-C_3_N_4_ and **d** top-down g-C_3_N_4_ membranes. **e** The DFT calculations about stacking states of bottom-up g-C_3_N_4_ nanosheets in g-C_3_N_4_ membranes. The front represents the first layer of nanosheet in a two-layer system of g-C_3_N_4_ and the rear represents another layer of nanosheet below the first layer of nanosheet. **f** The AA stacking of bottom-up g-C_3_N_4_ nanosheets. C, N, and H atoms are colored in gray, blue, and white, respectively. **g** 1D and corresponding 2D wide-angle XRD patterns of bottom-up g-C_3_N_4_ nanosheets and g-C_3_N_4_ membrane. Source data are provided as a Source data file.

It has been reported that the guest solvent can tune the stacking modes of nanosheets according to host-guest noncovalent interactions, such as steric hindrance or van der Waals interactions^35^. Therefore, in this work, using isopropanol as a guest molecule to weaken the π-π interaction between g-C_3_N_4_ nanosheets to prevent undesired re-stacking, the lamellar g-C_3_N_4_ membranes were prepared on porous anodic aluminum oxide (AAO) substrates (Supplementary Fig. 9). All the prepared g-C_3_N_4_ membranes are intact with no detectable pinholes or cracks and have homogeneous elemental distributions, as seen in the SEM images (Fig. 2a, b and Supplementary Figs. 10–12). The cross-sectional transmission electron microscopy (TEM) image of the g-C_3_N_4_ membrane (Fig. 2c) reveals the turbostratic arrangement of the bottom-up nanosheets, which can be attributed to the stronger repulsive interactions weakening the π-π interactions between adjacent nanosheets (Supplementary Fig. 13). Such structures have also been reported in GO membranes^36^. While it is shown in Fig. 2d that the top-down g-C_3_N_4_ nanosheets form a compact membrane structure. Besides, considering that the aligned and unaligned stacking between adjacent layers will affect the separation applications of the membrane^37^, the stacking modes of g-C_3_N_4_ nanosheets were calculated by DFT. As shown in Fig. 2e, the calculated total energy of bilayer bottom-up g-C_3_N_4_ shows that its AA stacking has minimum energy configuration, indicating that the bottom-up g-C_3_N_4_ nanosheets tend to the aligned AA stacking in the g-C_3_N_4_ membrane, forming conceivable gas-permeable interlayer pathways (Fig. 2f). In contrast, the top-down g-C_3_N_4_ nanosheets tend to be nonaligned AB stacking (Supplementary Fig. 14), which greatly reduces the effective sieving channel of g-C_3_N_4_ membrane and thus blocks the transmission of gas. Moreover, the corrugated surfaces of the g-C_3_N_4_ membrane (Supplementary Fig. 15) suggest disordered stacked nanosheets, which is consistent with the TEM results. From the 1D and corresponding 2D XRD patterns of the g-C_3_N_4_ nanosheets and membrane (Fig. 2g), the (002) diffraction peak of the g-C_3_N_4_ membrane becomes broader than that of the nanosheets, further indicating the disordered stacking of the bottom-up nanosheets in the g-C_3_N_4_ membrane^38^. Grazing incidence angle X-ray diffraction could provide more accurate XRD information of the membrane by eliminating the interference of the substrates, which also shows a broad (002) diffraction peak with a disordered turbostratic arrangement in the g-C_3_N_4_ membrane (Supplementary Fig. 16). Indeed, such lamellar disordered structure may provide additional gas transport pathways and plays a dominant role in constructing ultra-permeable membranes. For example, Yang et al.^39^ reported that the performance of 2D MOF nanosheet membranes was correlated with the stacking order of the nanosheets, where the membrane with disordered stacking showed increased permeance and selectivity by 255% and 449% compared with that of ordered re-stacking, respectively. The XPS spectra of the g-C_3_N_4_ membrane confirms the preservation of the g-C_3_N_4_ structure in the membrane (Supplementary Fig. 17), where the characteristic peak of g-C_3_N_4_ membrane is similar to that of the prepared nanosheets. In addition, the g-C_3_N_4_ membrane exhibits the Young’s modulus up to 159 MPa, revealing its excellent mechanical property (Supplementary Fig. 18 and Supplementary Table 2).

### Gas separation performance of g-C_3_N_4_ membranes

**Fig. 3:**
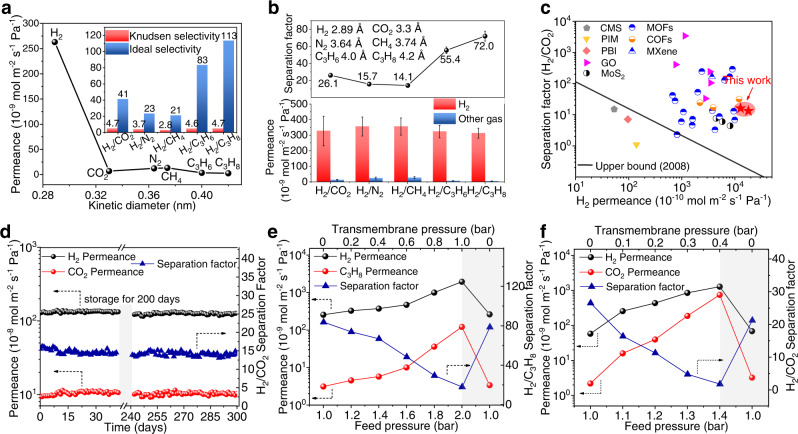
Gas separation performance through the g-C_3_N_4_ membranes. **a** Single gas permeation of the 1-μm-thick g-C_3_N_4_ membrane at room temperature and 1 bar. The inset shows the gas selectivity for H_2_ over other gases. **b** Permeance and separation factors of the 1-μm-thick g-C_3_N_4_ membranes in the equimolar mixed-gas permeation at room temperature and 1 bar. Inset shows the kinetic diameters of various gas molecules. Errors bars indicate the standard deviation of three measurements. **c** Comparison of H_2_/CO_2_ separation performance of g-C_3_N_4_ membranes with state-of-the-art membranes at room temperature. **d** Long-term stability of the g-C_3_N_4_ membrane for equimolar H_2_/CO_2_ mixture. The gray areas represent the membrane was stored in an ambient environment without the introduction of any protective gas at room temperature for 200 days. **e** Gas permeances and H_2_/C_3_H_8_ separation factor of the g-C_3_N_4_ membrane as a function of the feed pressure at room temperature. The gray areas show the performance of g-C_3_N_4_ membrane after releasing the pressure. **f** Gas permeances and H_2_/CO_2_ separation factor of the g-C_3_N_4_ membrane as a function of the feed pressure at room temperature. The gray areas show the performance of g-C_3_N_4_ membrane after releasing the pressure. Source data are provided as a Source data file.

The gas separation performance of the g-C_3_N_4_ membranes were measured systematically using Wicke-Kallenbach permeation cells (Supplementary Fig. 19). The H_2_ flux through a 1 μm-thick g-C_3_N_4_ membrane reaches high permeance of 3.3 × 10^−7^ mol m^−2^ s^−1^ Pa^−1^ (Fig. 3a, b and Supplementary Fig. 20). As the kinetic diameter of the molecules increases, the gas permeance decreases sharply, indicating a clear cut-off between H_2_ and the other tested gases. The H_2_ to CO_2_, N_2_, CH_4_, C_3_H_6_, and C_3_H_8_ selectivity are 41, 23, 21, 83, and 113, respectively, which far exceeds the Knudsen selectivity (Supplementary Table 3). However, the 1-μm-thick g-C_3_N_4_ membranes assembled by the top-down g-C_3_N_4_ nanosheets exhibit similar selectivity to the Knudsen diffusion (Supplementary Fig. 21). Considering the same material and thickness of the two types of membranes, their performance differences are attributed to membrane defects. The g-C_3_N_4_ membrane assembled by the bottom-up g-C_3_N_4_ nanosheets exhibits noticeable separation performance while the one using the top-down nanosheets does not, indicating that the defects have a significant adverse effect on membrane performance. Recently, Li et al.^40^ developed a strategy of vapor linker exchange inducing partial amorphization to conglutinate grain boundary/crack defects of membranes, where the ZIF-8 composite membrane showed competitive H_2_/CO_2_ selectivity up to 2400, which also indicated the great significance of restraining membrane defects, further illustrating the advantages of the bottom-up method for the synthesis of g-C_3_N_4_. Gas permeation through the g-C_3_N_4_ membrane is different from Knudsen diffusion and is mainly governed by the kinetic gas diameter rather than its molecular weight, known as the size exclusion or molecular sieving mechanism^41^ (Supplementary Fig. 22). Besides, the g-C_3_N_4_ membranes with different thicknesses were fabricated (Supplementary Fig. 23 and Supplementary Table 4). As the membrane thickness increases, the H_2_ permeance of the g-C_3_N_4_ membrane decreases and the H_2_/CO_2_ selectivity increases, which is characteristic of molecular separation membranes. The 300-nm-thick g-C_3_N_4_ membranes exhibit an H_2_ flux of 1.3 × 10^−6^ mol m^−2^ s^−1^ Pa^−1^ with an H_2_/CO_2_ separation factor of 16. The results are promising for industrial applications as enhancing permeance is more important than improving selectivity to reduce separation costs^42^. Indeed, the g-C_3_N_4_ membranes show ultra-high H_2_ flux with considerable selectivity compared to the other membranes reported in the literatures (see Supplementary Table 5), which breaks the conventional upper bound, as shown in Fig. 3c. Moreover, the g-C_3_N_4_ membrane exhibits excellent stability during a 1000 h continuous operation. After being stored in an ambient environment without the introduction of any protective gas at room temperature for 200 days, the membrane was examined again, whose H_2_/CO_2_ separation performance was still stable and as high as the fresh one as shown in Fig. 3d. Furthermore, the gas performance of the g-C_3_N_4_ membranes were further investigated in harsh environments, including water vapor, elevated temperatures and pressures (Supplementary Fig. 24)^43^. When exposed to a 3 vol% water vapor environment, the membrane can operate stably for 100 h at room temperature (Supplementary Fig. 25). After three temperature cycles between 25 and 150 °C, the separation performance of the g-C_3_N_4_ membrane is recovered (Supplementary Figs. 26–28), which can be attributed to the excellent thermostability of the g-C_3_N_4_ material^22^ (Supplementary Fig. 29). Additionally, the g-C_3_N_4_ membrane was also evaluated by durability testing with an equimolar H_2_/CO_2_ mixture feed at 120 °C in the presence of water vapor (water activity of 0.353, Supplementary Note 1). It can be seen that the g-C_3_N_4_ membrane performs well for 100 h even under wet gas mixture conditions at elevated temperatures (Supplementary Fig. 30), indicating the good hydrothermal stability of the g-C_3_N_4_ membrane. The stability of g-C_3_N_4_ system can also be confirmed by MD simulation. It can be seen that g-C_3_N_4_ system shows very small energy fluctuations in the long-term MD simulations (Supplementary Fig. 31). This is consistent with the substantially unchanged schematic diagrams of the g-C_3_N_4_ layer before and after MD simulations, also reflecting its good structural stability^44^ (Supplementary Fig. 32). Moreover, when the feed pressure increases to 2 bar (transmembrane pressure: 1 bar), the g-C_3_N_4_ membrane exhibits a decreased H_2_/C_3_H_8_ separation factor of 17, which can be recovered to the initial value basically after releasing the pressure, as shown in Fig. 3e. However, the H_2_ and CO_2_ permeances increase rapidly while the H_2_/CO_2_ selectivity decreases with the increased feed pressure (Fig. 3f). The possible reason behind the decrease in selectivity at high pressure might be the existence of the parallel non-selective transport pathways where viscous diffusion is prevalent and which dominates the membrane performance when the transmembrane pressure difference is greater than zero^45,46^.

## Discussion

**Fig. 4:**
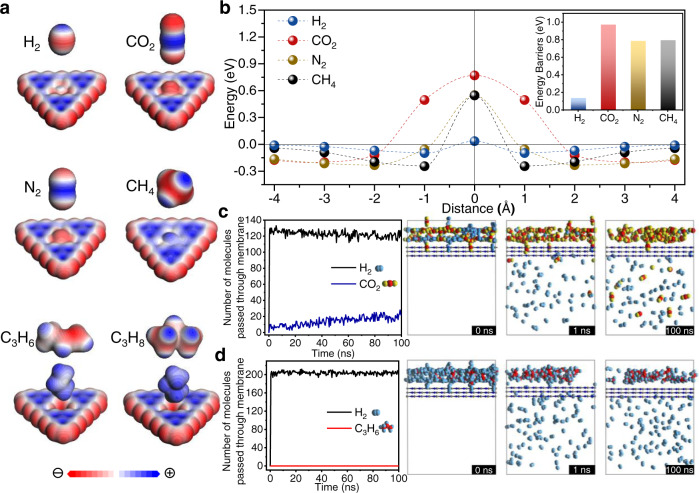
Gas separation mechanism of the g-C_3_N_4_ layer. **a** Schematic illustrations of gas molecules with electrostatic potentials distribution through the g-C_3_N_4_ layer. **b** Potential energy surfaces for H_2_, CO_2_, N_2_, and CH_4_ on the g-C_3_N_4_ layer. Inset is the energy barrier for gas molecules across the monolayer g-C_3_N_4_. The number of gas molecules of **c** H_2_/CO_2_ mixtures and **d** H_2_/C_3_H_6_ mixtures through the g-C_3_N_4_ layer in molecular dynamics (MD) simulations as a function of simulation time. Simulation snapshots at 0, 1, and 100 ns for H_2_/CO_2_ mixtures and H_2_/C_3_H_6_ mixtures are given. C, O, and H atoms are colored in red, yellow, and blue, respectively. Source data are provided as a Source data file.

The permeances of H_2_, CO_2_, N_2_, and CH_4_ do not completely follow the order of kinetic diameters of gas molecules, indicating gas transport behaviors are not only affected by molecular size. Therefore, the behaviors of gas molecules passing through the g-C_3_N_4_ layer were investigated using DFT. Considering the universality, we still used the most typical g-C_3_N_4_ structure to perform the simulation process. It is found that when H_2_, CO_2_, N_2_, and CH_4_ molecules pass through the g-C_3_N_4_ layer, the electron clouds of gas molecules partially overlap with the atoms around the g-C_3_N_4_, as shown in the partial density of states (PDOS)^47^ results (Supplementary Fig. 33). As a result, H_2_, CO_2_, N_2_, and CH_4_ molecules can pass through g-C_3_N_4_ layer after the partial overlap of electron clouds of the gas molecules and the g-C_3_N_4_. However, in virtue of a large number of negatively polarized N atoms in the g-C_3_N_4_ nanosheets (red reflects negatively polarized sites, blue represents the opposite), g-C_3_N_4_ nanosheets show stronger electrostatic interactions with CO_2_ molecules with the deepest blue^48^, which increases the resistance to CO_2_ diffusion and results in a high separation factor of H_2_/CO_2_ (Fig. 4a). While CH_4_ with light blue possesses relatively weak interactions with g-C_3_N_4_ nanosheets, resulting in relatively fast CH_4_ diffusion. This is consistent with N-functionalized graphene membranes reported previously^49^. When the carbon atoms were substituted by nitrogen in the porous graphene membrane, the properties of the nanosheets changed and influenced the gas permeability. Consequently, the calculated energy barriers (E_*b*_)^18^ for gas molecules across the nanosheets are 0.132, 0.969, 0.782, and 0.791 eV for H_2_, CO_2_, N_2_, and CH_4_, respectively (Fig. 4b and Supplementary Table 6), which suggests that g-C_3_N_4_ is promising for sieving H_2_ from these larger molecules, especially CO_2_. The adsorption isotherms of H_2_, CO_2_, N_2_, and CH_4_ on the g-C_3_N_4_ membranes at room temperature also indicate that the g-C_3_N_4_ membranes tend to preferentially adsorb CO_2_ compared to other gases, such as H_2_, N_2_, CH_4_ (Supplementary Fig. 34), delaying the CO_2_ transport^50^. However, the permeation of CH_4_ is slightly higher than that of N_2_ in the experiment, which is different from the simulation results. This may be because the deviation in the experimental measurement or CH_4_ as a spherical molecule^51^ is easier to pass through. It should be noted that the g-C_3_N_4_ membranes exhibit a sharp separation of H_2_/C_3_ with higher gas selectivity for H_2_/C_3_H_6_ and H_2_/C_3_H_8_ (exceeding 80), which can be attributed to the size sieving effect due to the larger size of C_3_H_6_ (4.0 Å) and C_3_H_8_ (4.2 Å) molecules^52^. These results show that the gas separation mechanism of the g-C_3_N_4_ membrane is based on the synergistic effect of size exclusion and the interactions between gas molecules and g-C_3_N_4_ nanosheets.

Moreover, MD simulations were conducted to study the gas transport through the g-C_3_N_4_ membrane. In H_2_/CO_2_ mixed gas simulations (Fig. 4c), it is found that all H_2_ molecules have moved quickly through the g-C_3_N_4_ layers at the very beginning, yielding a diffusivity ratio of 130:4 for H_2_:CO_2_ with the H_2_/CO_2_ selectivity of 32.5 at 1.0 ns. The predicted selectivity decreases with extended time because there is no H_2_ molecule on the feed side while CO_2_ continues to pass through in the subsequent simulation. Besides, the selectivity predicted by the DFT and MD simulations shows several orders of magnitude differences (see Supplementary Note 2), which may be that DFT calculations use different reference states for the energies (separate nucleus, electrons) while potential energy with classical MD simulation only includes the intra and intermolecular potential energies and not the piece due to internal part of the molecular partition function. There is a great difference between the two calculation methods. Therefore, in this work, there is not a strict one-to-one correspondence between the results by DFT calculations and MD simulations, as reported by Wang et al.^53^. Even so, the MD results qualitatively agree well with the DFT results. With respect to H_2_/C_3_H_6_ mixtures (Fig. 4d), H_2_ quickly passes through the g-C_3_N_4_ layer while C_3_H_6_ does not, exhibiting a sharpening sieving effect for H_2_/C_3_H_6_.

In this work, lamellar g-C_3_N_4_ nanosheet membranes with excellent gas separation performances are successfully constructed. The membranes are assembled using high-quality nanosheets obtained through a bottom-up method, allowing the exfoliation step to be skipped, thus maintaining structural integrity. Guest molecules are introduced during membrane preparation to prevent re-stacking of the g-C_3_N_4_ nanosheets. The fabricated laminar g-C_3_N_4_ nanosheet membranes exhibit H_2_ permeance of up to 1.3 × 10^−6^ mol m^−2^ s^−1^ Pa^−1^ due to the synergistic effect of the high-density sieving channels and disordered stacking structure. In addition, the gas separation mechanism of the g-C_3_N_4_ membrane is based on the integrated gating effects of size exclusion and the interactions between gas molecules and g-C_3_N_4_ nanosheets, as evidenced by both the experimental results and computational simulations. These membranes also show good stability even under harsh practice-relevant environments. The excellent separation performance with long-term stability enables the g-C_3_N_4_ membrane to serve as a promising candidate for H_2_ purification, offering an opportunity for the development of carbon neutrality.

## Methods

### Materials

Melamine (99%, Aladdin), phosphoric acid (≥85%, Guangzhou Chemical Reagent Factory), isopropanol (Guangzhou Chemical Reagent Factory), ethanol (Runjie Chemistry), glycerol (Runjie Chemistry), AAO substrate with a diameter of 15 mm (with a pore size of 160–200 nm, PuYuan Nanotechnology Limited Company). All of the materials were used as purchased without further purification.

### Preparation of the g-C_3_N_4_ nanosheets

First, a bottom-up method was employed to fabricate the g-C_3_N_4_ nanosheets^25^, as follows. Melamine (1 g) and phosphoric acid (1.2 g) were dissolved in 100 mL of deionized water at 80 °C in a thermostatic water bath until the melamine dissolved completely. Then the solution was transferred into a hydrothermal reactor and heated at 180 °C for 10 h. After centrifugation at 2795 × *g* for 30 min, the obtained mixture was dried in an oven at 60 °C. The layered precursors were obtained and then refluxed for 3 hours at 90 °C with a 20 mL mixed solution of ethanol and glycerol (3:1, v-v). The powders were washed with ethanol several times, and then the layered precursors after intercalation were obtained after dried at 60 °C. Here, to keep the inherent nanoporous structures of g-C_3_N_4_, we chose N_2_ to achieve an inert operation condition. It has been reported that the gas atmosphere affects the nanoporous structures of g-C_3_N_4_, and more complete g-C_3_N_4_ nanoporous structures were obtained in an inert atmosphere.^54^ Aminabhavi et al.^55^ found that in the process of synthesizing g-C_3_N_4_, ammonia was obtained in melamine decomposition and made g-C_3_N_4_ produce N-defects. So the layered precursors after intercalation were heated to 520 °C under N_2_ condition with the heating rate of 2 °C min^−1^ and kept for 2 h. Then high-quality g-C_3_N_4_ nanosheets of 6–8 mg can be obtained. For comparison, a traditional top-down method was also employed to fabricate the g-C_3_N_4_ nanosheets^56^. 10 g melamine was heated to 520 °C for 6.5 h under an air atmosphere with a heating and cooling speed of 5 °C min^−1^. Then the obtained g-C_3_N_4_ powders (1 mg) were dispersed in the water (50 mL) to obtain the g-C_3_N_4_ suspension at the concentration of 0.02 mg mL^−1^. The g-C_3_N_4_ nanosheets suspension was obtained by ultrasonic treatment for 2 h.

### Preparation of the g-C_3_N_4_ membranes

For assembling nanosheets into membranes by vacuum filtration, the g-C_3_N_4_ nanosheets powders (1 mg) prepared by the bottom-up method need to be dispersed in isopropanol (50 mL) to obtain the nanosheets suspension. The nanosheets suspension was treated by ultrasonication for 5 min for better dispersion. Then the g-C_3_N_4_ membranes were fabricated on AAO substrates by filtering a certain amount of two different g-C_3_N_4_ nanosheets suspensions, respectively. Next, these g-C_3_N_4_ membranes were placed in a vacuum drying chamber at room temperature for more than 12 h to remove residual solvents in the membranes.

### Gas permeation test

All the gas permeation tests were performed in a homemade Wicke-Kallenbach apparatus^41^. The gases with different kinetic diameters were used as the feed gas, while Ar was used as the sweep gas. The gas volumetric flow rate was constantly controlled at 50 mL min^−1^ for single and mixed gas tests. Gas chromatography (GC Agilent 7890) was employed to obtain the gas concentrations of permeate gas. The gas flow was controlled using mass flow controllers (MFCs) and corrected by a bubble flowmeter. The membrane module was packed with heating tape, and thermocouple and temperature controller devices were used to control the temperature and heating rate (2 °C min^−1^). Feed gas was saturated with water vapor before feeding to the membrane module. The partial pressure of water vapor in the feed gas was varied by controlling the temperature of the water tank. The permeate stream was chilled in an iced cold trap. The relative humidity (RH) of the feed stream was measured by a humidity transmitter.

At equilibrium conditions, the water activity (*a*_w_) is calculated by Eq. (1):

1 $${a}_{{{{{{\rm{w}}}}}}}=\frac{{{{{{\rm{RH}}}}}}}{100}=\frac{{P}_{{{{{{{\rm{H}}}}}}}_{2}{{{{{\rm{O}}}}}}}}{{P}_{{{{{{\rm{sat}}}}}}}}$$

where ${P}_{{{{{{{\rm{H}}}}}}}_{2}{{{{{\rm{O}}}}}}}$ is the water vapor partial pressure, ${P}_{{{{{{\rm{sat}}}}}}}$ is the saturation water vapor pressure at the stream temperature and pressure^57^.

The following equations calculate the gas permeance (*P*_i_, mol m^−2^ s^−1^ Pa^−1^) and ideal selectivity *S*_i/j_,

2 $${P}_{{{{{{\rm{i}}}}}}}=\frac{{N}_{{{{{{\rm{i}}}}}}}}{{\Delta P}_{{{{{{\rm{i}}}}}}}\cdot A}$$

3 $${S}_{{{{{{\rm{i}}}}}}/{{{{{\rm{j}}}}}}}=\frac{{P}_{{{{{{\rm{i}}}}}}}}{{P}_{{{{{{\rm{j}}}}}}}}$$

where *N*_i_ (mol s^−1^) is the permeate flow rate of the component gas i, Δ*P*_i_ (Pa) is the transmembrane pressure difference of i, and *A* (m^2^) is the membrane area.

The mixed gas separation factor (*α*_i/j_) is calculated as follows:

4 $${\alpha }_{{{{{{\rm{i}}}}}}/{{{{{\rm{j}}}}}}}=\frac{{y}_{{{{{{\rm{i}}}}}}}/{y}_{{{{{{\rm{j}}}}}}}}{{x}_{{{{{{\rm{i}}}}}}}/{x}_{{{{{{\rm{j}}}}}}}}$$

where *x*_i_ and *x*_j_ are the volumetric fractions of component i and component j at the feed side, respectively; *y*_i_ and *y*_j_ are corresponding volumetric fractions at the permeate side.

### DFT simulations

All the computational simulations were performed using the Materials Studio 7.0 package^58^. To determine the stable stacking status mode of nanosheets in g-C_3_N_4_ membranes, we constructed a two-layer system of g-C_3_N_4_ corresponding to both of them. One layer shifted to specific extents related to the other layer. The total energy of these two-layer systems is calculated in the Forcite module. The van der Waals (vdW) interactions between layers are described by Dreiding force field^59^, with cubic spline and a cutoff distance of 12.0 Å for vdW truncation. The electrostatic interactions are modeled by Ewald^60^ for the summation of atomic charges of g-C_3_N_4_ calculated using the QEq method, with the accuracy set to be 1 × 10^−4^ kcal/mol. The framework of g-C_3_N_4_ is assumed to be rigid in the calculations.

The PDOS analysis of g-C_3_N_4_ and gas molecules (H_2_, CO_2_, N_2_, and CH_4_) were computed by GGA/PBE level of functional under DNP basis set in Dmol^3^ module^61^, where DFT-D correction was considered with Grimme method^62^, and the core electrons were treated by an all-electron method. During the simulations, 1.0 × 10^−6 ^Ha of self-consistent field (SCF) tolerance and 500 SCF cycles were used, and 0.05 Ha of thermal smearing were applied to orbital occupation to speed up convergence. The periodic boundary conditions were considered in DFT calculations.

With the same simulation level of PDOS, the electrostatic potential distributions of gas-free g-C_3_N_4_ and gas molecules (H_2_, CO_2_, N_2_, CH_4_, C_3_H_6_, and C_3_H_8_) were computed. In addition, the potential energy surfaces were obtained by calculating the interaction energies between gas molecules and g-C_3_N_4_ nanosheets at different distances from the nanosheets. Typically, a gas molecule was placed at several positions on the nanosheets, and the mass center of the gas was fixed in the z-direction (perpendicularly to the g-C_3_N_4_ layer). The interaction energy (*E*_int_) of the gas molecules with g-C_3_N_4_ at the corresponding position is calculated by Eq. (5).

5 $${E}_{{{{{{\rm{int}}}}}}}={E}_{{{{{{{\rm{g}}}}}}-{{{{{\rm{C}}}}}}}_{3}{{{{{{\rm{N}}}}}}}_{4}+{{{{{\rm{gas}}}}}}}-\left({E}_{{{{{{{\rm{g}}}}}}-{{{{{\rm{C}}}}}}}_{3}{{{{{{\rm{N}}}}}}}_{4}}+{E}_{{{{{{\rm{gas}}}}}}}\right)$$

where ${E}_{{{{{{{\rm{g}}}}}}-{{{{{\rm{C}}}}}}}_{3}{{{{{{\rm{N}}}}}}}_{4}+{{{{{\rm{gas}}}}}}}$ is the total energy of the g-C_3_N_4_-gas configuration, and $\,{E}_{{{{{{{\rm{g}}}}}}-{{{{{\rm{C}}}}}}}_{3}{{{{{{\rm{N}}}}}}}_{4}}$ and ${E}_{{{{{{\rm{gas}}}}}}}$ are the single point energy of g-C_3_N_4_ and the gas molecules, respectively.

Next, the *E*_b_ (energy barrier) is defined as the difference between the interaction energies at the transition state (TS, z = 0) and the stable state (SS, the stable adsorption state when the attractive interaction is maximum and z ≠ 0) of the gas molecule on g-C_3_N_4_^26^.

6 $${E}_{{{{{{\rm{b}}}}}}}={E}_{{{{{{\rm{TS}}}}}}}-{E}_{{{{{{\rm{SS}}}}}}}$$

where *E*_TS_ and *E*_SS_, respectively, stand for the TS energy and SS energy when a gas molecule permeates through the g-C_3_N_4_ nanosheet. This modeling method has been successfully used to explore the interaction mechanism between porous materials and small gas molecules^26^.

### MD simulations

All the computational simulations were performed using the Materials Studio 7.0 package^58^. The gas permeation of H_2_/CO_2_ and H_2_/C_3_H_6_ mixtures through the g-C_3_N_4_ layer was modeled by MD simulations. For H_2_/CO_2_ mixtures, 260 gas molecules (130 for each gas species) were placed in the feed chamber in the simulation. For H_2_/C_3_H_6_ mixtures, 420 gas molecules (210 for each gas species) were placed in the feed chamber. Then gas mixtures were loaded into the two-layer system of g-C_3_N_4_ employing the Sorption module with the Metropolis method, where 1 × 10^6^ kPa of pressure is fixed for each gas. The Dreiding force field and interaction settings were the same as the energy calculations of the gas-free g-C_3_N_4_ system. The loaded gas molecules were then optimized with a Forcite module for energy minimization of the adsorbent-adsorbate system, while the atom of g-C_3_N_4_ was kept rigid. Subsequently, MD simulations were performed with NVT ensemble in the Forcite module at 298 K, where 1 × 10^5^ ps of total simulation time was used with a time step of 1.0 fs. The Berendsen method^63^ was applied to maintain the temperature. During the MD simulation, the Dreiding force field and Ewald summation describe the vdW and electrostatic interaction between the gas and g-C_3_N_4_. The periodic boundary conditions were considered in three dimensions. MD simulations were run for 10,000 ps to study the potential energy fluctuations of the g-C_3_N_4_ system with 298 K temperature to verify the stability of the g-C_3_N_4_ system.

### Characterization

NMR measurements were recorded on an AVANCE III spectrometer (Bruker) operating at a proton frequency of 400 MHz. XRD patterns were recorded under ambient conditions with a Bruker D8 Advance diffractometer with Cu Kα radiation at 40 kV and 40 mA. The two-dimensional XRD patterns of the g-C_3_N_4_ membrane and g-C_3_N_4_ powder were obtained by XRD (Rigaku Smart Lab X-Ray Diffractometer) equipped with a 2D X-ray detector using Cu Kα radiation source from 5° to 40° under two-dimensional detection mode. The microstructure of the membranes was observed by the SEM using a HITACHI SU8200. The AFM images were obtained using a Bruker MultiMode 8 scanning probe microscope (SPM, VEECO) in the tapping mode. The room-temperature EPR spectra were measured with Bruker A300. The XPS analysis was performed using an ESCALAB 250 spectrometer (Thermo Fisher Scientific) with monochromated Al Kα radiation (1486.6 eV) under a 2 × 10^−9^ Torr pressure. The concentrations of the g-C_3_N_4_ nanosheets dispersions were measured by UV-vis spectrum (Shimadzu UV-2450). TEM images were obtained using a JEOL JEM-2100F microscope with an acceleration voltage of 200 kV. FTIR (Nicolet 5700 spectrometer) was used to detect the characteristic stretching vibration modes of the sample. Elemental analysis was measured on a Vario EL cube elementary. Thermogravimetric (TG) measurement was analyzed on a Netzsch STA 449F3 instrument under the flow of N_2_. The adsorption isotherms of H_2_, CO_2_, N_2_, and CH_4_ on the g-C_3_N_4_ membranes were measured using a Micromeritics (ASAP 2460) instrument. The mechanical properties were measured by using an Instron-5565 universal tensile testing machine (USA).

## Supplementary information

Supplementary Information

Peer Review File

## Data Availability

Further data that support the findings of this study are available on request from the corresponding author. Source data are provided with this paper.

## Source data

Source Data

## References

[1] Quadrelli R, Peterson S (2007). The energy-climate challenge: recent trends in CO_2_ emissions from fuel combustion. Energy Policy.

[2] Haszeldine RS (2009). Carbon capture and storage: how green can black be?. Science.

[3] Kenarsari SD (2013). Review of recent advances in carbon dioxide separation and capture. RSC Adv..

[4] IPCC. *IPCC Third Assessment Report Climate Change 2001* (Intergovernmental Panel on Climate Change, 2001).

[5] de Jongh PE (2011). Hydrogen storage keeping out the oxygen. Nat. Mater..

[6] Sen M, Dana K, Das N (2018). Development of LTA zeolite membrane from clay by sonication assisted method at room temperature for H_2_-CO_2_ and CO_2_-CH_4_ separation. Ultrason. Sonochem..

[7] Sholl DS, Lively RP (2016). Seven chemical separations to change the world. Nature.

[8] Gin DL, Noble RD (2011). Designing the next generation of chemical separation membranes. Science.

[9] Park HB, Kamcev J, Robeson LM, Elimelech M, Freeman BD (2017). Maximizing the right stuff: the trade-off between membrane permeability and selectivity. Science.

[10] Moghadam F, Park HB (2018). Two-dimensional materials: an emerging platform for gas separation membranes. Curr. Opin. Chem. Eng..

[11] Kim S, Wang HT, Lee YM (2019). 2D nanosheets and their composite membranes for water, gas, and ion separation. Angew. Chem. Int. Ed..

[12] Liu GP, Jin WQ, Xu NP (2016). Two-dimensional-material membranes: a new family of high-performance separation membranes. Angew. Chem. Int. Ed..

[13] Yang H (2019). Covalent organic framework membranes through a mixed-dimensional assembly for molecular separations. Nat. Commun..

[14] Cao KT (2015). Highly water-selective hybrid membrane by incorporating g-C_3_N_4_ nanosheets into polymer matrix. J. Membr. Sci..

[15] Cui YQ (2021). Emerging graphitic carbon nitride-based membranes for water purification. Water Res..

[16] Tian ZZ (2016). Enhanced gas separation performance of mixed matrix membranes from graphitic carbon nitride nanosheets and polymers of intrinsic microporosity. J. Membr. Sci..

[17] Soto-Herranz M (2018). Effects of protonation, hydroxylamination, and hydrazination of g-C_3_N_4_ on the performance of Matrimid (R)/g-C_3_N_4_ membranes. Nanomaterials.

[18] Ji YJ (2016). Heptazine-based graphitic carbon nitride as an effective hydrogen purification membrane. RSC Adv..

[19] Guo Y, Tang CM, Wang XB, Wang C, Fu L (2019). Density functional calculations of efficient H_2_ separation from impurity gases (H_2_, N_2_, H_2_O, CO, Cl_2_, and CH_4_) via bilayer g-C_3_N_4_ membrane. Chin. Phys. B.

[20] Wang XR (2017). Reversed thermo-switchable molecular sieving membranes composed of two-dimensional metal-organic nanosheets for gas separation. Nat. Commun..

[21] Li R (2019). Graphitic carbon nitride (g-C_3_N_4_) nanosheets functionalized composite membrane with self-cleaning and antibacterial performance. J. Hazard. Mater..

[22] Niu P, Zhang LL, Liu G, Cheng HM (2012). Graphene-like carbon nitride nanosheets for improved photocatalytic activities. Adv. Funct. Mater..

[23] Xu J, Zhang LW, Shi R, Zhu YF (2013). Chemical exfoliation of graphitic carbon nitride for efficient heterogeneous photocatalysis. J. Mater. Chem. A.

[24] Wang YJ (2017). Water transport with ultralow friction through partially exfoliated g-C_3_N_4_ nanosheet membranes with self-supporting spacers. Angew. Chem. Int. Ed..

[25] Xiao YT (2019). Molecule self-assembly synthesis of porous few-layer carbon nitride for highly efficient photoredox catalysis. J. Am. Chem. Soc..

[26] Villalobos LF (2020). Large-scale synthesis of crystalline g-C_3_N_4_ nanosheets and high-temperature H_2_ sieving from assembled films. Sci. Adv..

[27] Seyfarth L, Seyfarth J, Lotsch BV, Schnick W, Senker J (2010). Tackling the stacking disorder of melon-structure elucidation in a semicrystalline material. Phys. Chem. Chem. Phys..

[28] Lotsch BV (2007). Unmasking melon by a complementary approach employing electron diffraction, solid-state NMR spectroscopy, and theoretical calculations-structural characterization of a carbon nitride polymer. Chem. Eur. J..

[29] Hu YC, Shim Y, Oh J, Park S, Ishii Y (2017). Synthesis of ^13^C-,^15^N-labeled graphitic carbon nitrides and NMR-based evidence of hydrogen-bonding assisted two-dimensional assembly. Chem. Mater..

[30] Kessler FK (2017). Functional carbon nitride materials design strategies for electrochemical devices. Nat. Rev. Mater..

[31] Zhao HX (2014). Atomic single layer graphitic-C_3_N_4_: fabrication and its high photocatalytic performance under visible light irradiation. RSC Adv..

[32] Liang QH, Li Z, Huang ZH (2015). Holey graphitic carbon nitride nanosheets with carbon vacancies for highly improved photocatalytic hydrogen production. Adv. Funct. Mater..

[33] Xiao K, Giusto P, Wen LP, Jiang L, Antonietti M (2018). Nanofluidic ion transport and energy conversion through ultrathin free-standing polymeric carbon nitride membranes. Angew. Chem. Int. Ed..

[34] Lv C (2018). Defect engineering metal-free polymeric carbon nitride electrocatalyst for effective nitrogen fixation under ambient conditions. Angew. Chem. Int. Ed..

[35] Tang WQ (2021). Controlling the stacking modes of metal-organic framework nanosheets through host-guest noncovalent interactions. Angew. Chem. Int. Ed..

[36] Kim HW (2013). Selective gas transport through few-layered graphene and graphene oxide membranes. Science.

[37] Ying YP (2020). Ultrathin two-dimensional membranes assembled by ionic covalent organic nanosheets with reduced apertures for gas separation. J. Am. Chem. Soc..

[38] Jang J (2020). Turbostratic nanoporous carbon sheet membrane for ultrafast and selective nanofiltration in viscous green solvents. J. Mater. Chem. A.

[39] Peng Y (2014). Metal-organic framework nanosheets as building blocks for molecular sieving membranes. Science.

[40] Su PC, Tang HY, Jia MM, Lin YS, Li WB (2022). Vapor linker exchange of partially amorphous metal-organic framework membranes for ultra-selective gas separation. AIChE J..

[41] Ding L (2018). MXene molecular sieving membranes for highly efficient gas separation. Nat. Commun..

[42] Merkel TC, Lin HQ, Wei XT, Baker R (2010). Power plant post-combustion carbon dioxide capture: an opportunity for membranes. J. Membr. Sci..

[43] Lei LF (2021). Carbon hollow fiber membranes for a molecular sieve with precise-cutoff ultramicropores for superior hydrogen separation. Nat. Commun..

[44] Malyi OI, Sopiha KV, Persson C (2019). Energy, phonon, and dynamic stability criteria of two-dimensional materials. ACS Appl. Mater. Inter..

[45] Wang B, Sun C, Zhou R, Xing W (2022). A super-permeable and highly-oriented SAPO-34 thin membrane prepared by a green gel-less method using high-aspect-ratio nanosheets for efficient CO_2_ capture. Chem. Eng. J..

[46] Song SC (2019). Preparation of SSZ-13 membranes with enhanced fluxes using asymmetric alumina supports for N_2_/CH_4_ and CO_2_/CH_4_ separations. Sep. Purif. Technol..

[47] Lawson M (2019). First-principles study of carbon capture and storage properties of porous MnO_2_ octahedral molecular sieve OMS-5. Powder Diffr..

[48] Piquero-Zulaica I (2019). Electron transmission through coordinating atoms embedded in metal-organic nanoporous networks. Phys. Rev. Lett..

[49] Shan MX (2012). Influence of chemical functionalization on the CO_2_/N_2_ separation performance of porous graphene membranes. Nanoscale.

[50] Liu H (2014). A hybrid absorption-adsorption method to efficiently capture carbon. Nat. Commun..

[51] Chi CL (2016). Facile preparation of graphene oxide membranes for gas separation. Chem. Mater..

[52] Li JR, Kuppler RJ, Zhou HC (2009). Selective gas adsorption and separation in metal-organic frameworks. Chem. Soc. Rev..

[53] Deng SW, Hu H, Zhuang GL, Zhong X, Wang JG (2018). A strain-controlled C_2_N monolayer membrane for gas separation in PEMFC application. Appl. Surf. Sci..

[54] Niu P, Yin LC, Yang YQ, Liu G, Cheng HM (2014). Increasing the visible light absorption of graphitic carbon nitride (melon) photocatalysts by homogeneous self-modification with nitrogen vacancies. Adv. Mater..

[55] Song XP, Yang Q, Jiang XH, Yin MY, Zhou LM (2017). Porous graphitic carbon nitride nanosheets prepared under self-producing atmosphere for highly improved photocatalytic activity. Appl. Catal. B Environ..

[56] Liu LF, Zhou YS, Xue J, Wang HH (2019). Enhanced antipressure ability through graphene oxide membrane by intercalating g-C_3_N_4_ nanosheets for water purification. AIChE J..

[57] Chen GQ, Scholes CA, Qiao GG, Kentish SE (2011). Water vapor permeation in polyimide membranes. J. Membr. Sci..

[58] Segall, M. et al. Materials Studio 7.0 (Accelrys, 2010).

[59] Li G (2017). Temperature-regulated guest admission and release in microporous materials. Nat. Commun..

[60] Ewald PP (1921). Die Berechnung optischer and elektrostatischer Gitterpotentiale. Ann. Phys..

[61] Delley B (2000). From molecules to solids with the DMol^3^ approach. J. Chem. Phys..

[62] Grimme S, Antony J, Ehrlich S, Krieg H (2010). A consistent and accurate ab initio parametrization of density functional dispersion correction (DFT-D) for the 94 elements H-Pu. J. Chem. Phys..

[63] Berendsen HJC, Postma JPM, van Gunsteren WF, DiNola A, Haak JR (1984). Molecular dynamics with coupling to an external bath. J. Chem. Phys..

