# Author Correction: Fast hydrogen purification through graphitic carbon nitride nanosheet membranes

**DOI:** 10.1038/s41467-022-34199-4

**Published:** 2022-10-25

**Authors:** Yisa Zhou, Ying Wu, Haoyu Wu, Jian Xue, Li Ding, Rui Wang, Haihui Wang

**Affiliations:** 1 School of Chemistry and Chemical Engineering, Guangdong Provincial Key Lab of Green Chemical Product Technology, South China University of Technology, Guangzhou, 510640 China; 2 Beijing Key Laboratory of Membrane Materials and Engineering, Department of Chemical Engineering, Tsinghua University, Beijing, 100084 China

**Keywords:** Chemical engineering, Two-dimensional materials, Two-dimensional materials, Structural properties

Correction to: *Nature Communications* 10.1038/s41467-022-33654-6, published online 04 October 2022

The original version of this Article contained errors in Fig. 2a, Fig. 2b, and Fig. 3d.

The scale bars in Fig. 2a and Fig. 2b were incorrectly labelled as ‘1 µm’ and the membrane thicknesses were omitted. The correct version of the scale bars in Fig. 2a and Fig. 2b is ‘2 µm’ and the membrane thicknesses were indicated as ‘1 µm’.

The X-axis in Fig. 3d was labelled incorrectly as ‘Hours (h)’. The correct version of the X-axis label in Fig. 3d is ‘Time (days)’.

The original version of this Article also contained errors in the second, third, and fourth sentences of the ‘Gas separation performance of g-C_3_N_4_ membranes’ section of the Results, in which Fig. 3b was cited in the wrong position.

The original, incorrect text read ‘The H2 flux through a 1 μm-thick g-C_3_N_4_ membrane reaches high permeance of 3.3 × 10^−7^ mol m^−2^ s^−^^1^ Pa^−1^ (Fig. 3a and Supplementary Fig. 20). As the kinetic diameter of the molecules increases, the gas permeance decreases sharply, indicating a clear cut-off between H_2_ and the other tested gases. The H_2_ to CO_2_, N_2_, CH_4_, C_3_H_6_, and C_3_H_8_ selectivity are 41, 23, 21, 83, and 113, respectively, which far exceeds the Knudsen selectivity (Fig. 3b and Supplementary Table 3)’.

The correct version states ‘The H_2_ flux through a 1 μm-thick g-C_3_N_4_ membrane reaches high permeance of 3.3 × 10^−7^ mol m^−2^ s^−1^ Pa^−1^ (Fig. 3a, b and Supplementary Fig. 20). As the kinetic diameter of the molecules increases, the gas permeance decreases sharply, indicating a clear cut-off between H_2_ and the other tested gases. The H_2_ to CO_2_, N_2_, CH_4_, C_3_H_6_, and C_3_H_8_ selectivity are 41, 23, 21, 83, and 113, respectively, which far exceeds the Knudsen selectivity (Supplementary Table 3).’

These have been corrected in both the PDF and HTML versions of the Article.

