# Supplementary material for: Fast hydrogen purification through graphitic carbon nitride nanosheet membranes
Source: Nat Commun. 2022 Oct 4;13:5852. doi: 10.1038/s41467-022-33654-6 (PMC9532387; doi:10.1038/s41467-022-33654-6)
Supplement: Supplementary file 1 — Supplementary Information [file 41467_2022_33654_MOESM1_ESM.pdf]

Supporting Information

Fast hydrogen purification through graphitic carbon nitride nanosheet membranes

Yisa Zhou[1,3], Ying Wu[1,3], Haoyu Wu[2], Jian Xue[1✉], Li Ding[1], Rui Wang[1], and Haihui Wang[2✉]

[1]School of Chemistry and Chemical Engineering, Guangdong Provincial Key Lab of Green Chemical Product Technology, South China University of Technology, Guangzhou 510640, China.

[2]Beijing Key Laboratory of Membrane Materials and Engineering, Department of Chemical Engineering, Tsinghua University, Beijing 100084, China.

[3]These authors contributed equally: Yisa Zhou, Ying Wu.

✉e-mail: xuejian@scut.edu.cn; cehhwang@tsinghua.edu.cn

1. Supplementary Figures(S1-S34) **2-36**

2. Supplementary Tables(S1-S6) **37-42**

3. Supplementary References **43-47**

1. Supplementary Figures

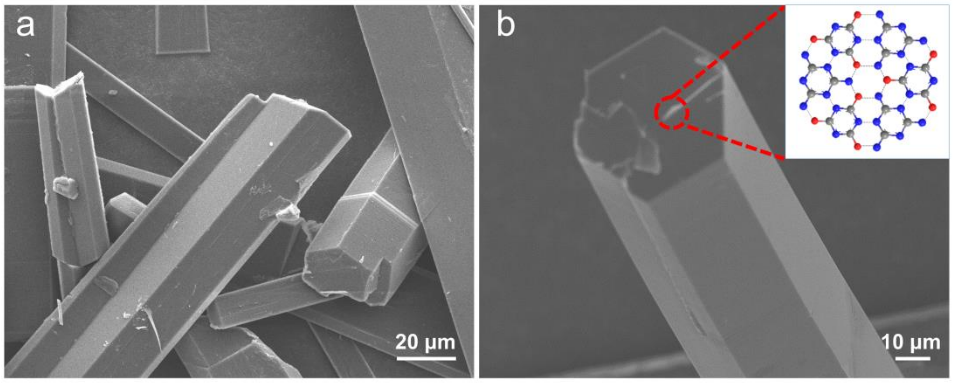

Supplementary Fig. 1. SEM images of the layered precursors formed by self-assembly of melamine and cyanuric acid. Inset is the schematic diagram of the layered precursor. C: gray; N: blue; O: red.

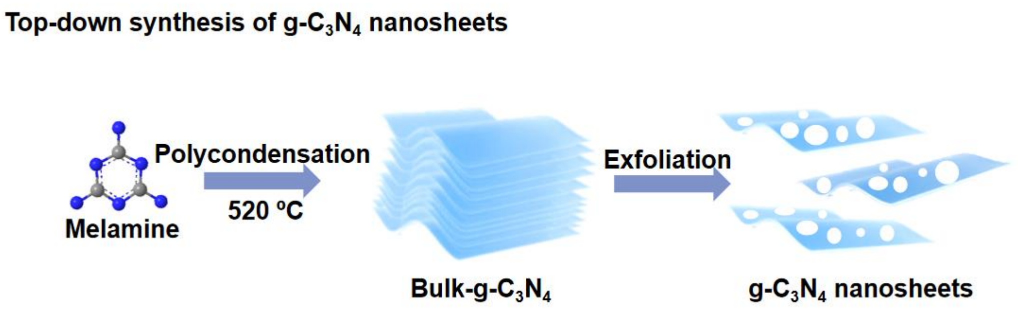

Supplementary Fig. 2. The top-down fabrication process of g-C_3N_4 nanosheets. C: gray; N: blue.

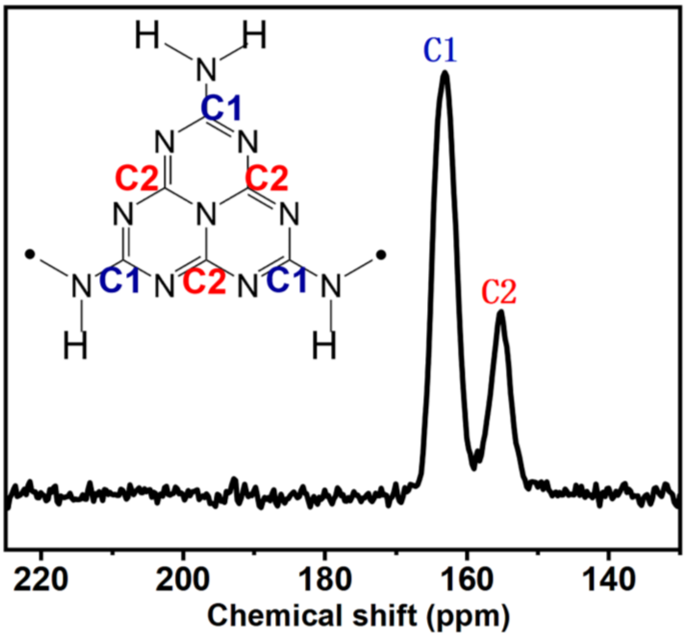

Supplementary Fig. 3. The ^{13}C solid-state NMR spectrum of bottom-up g-C_3N_4 nanosheets.

The peak heights of C1 and C2 carbon should be should be equal in ^{13}C NMR spectra, theoretically. However, the peak heights of C1 and C2 carbon are not equal, which is probably because the ^{13}C spectra obtained by the ^{1}H-^{13}C CP-MAS NMR technique cannot provide information regarding the relative abundance of carbon atoms in different chemical environments according to the signal strength, but rather record the diversity in CP efficiencies[1]. Actually, the signal intensity ratio varies due to different proximities to ^{1}H species in the ^{1}H-^{13}C cross polarization/MAS (CP-MAS) mode. The peak of C1 shows higher intensity, which because that C1 is closer to protons of nonpolymerized NH_2 or partially polymerized NH species, where ^{13}C spin polarized NMR signals are greatly enhanced from neighboring ^{1}H species via a ^{1}H-^{13}C dipolar coupling[2].

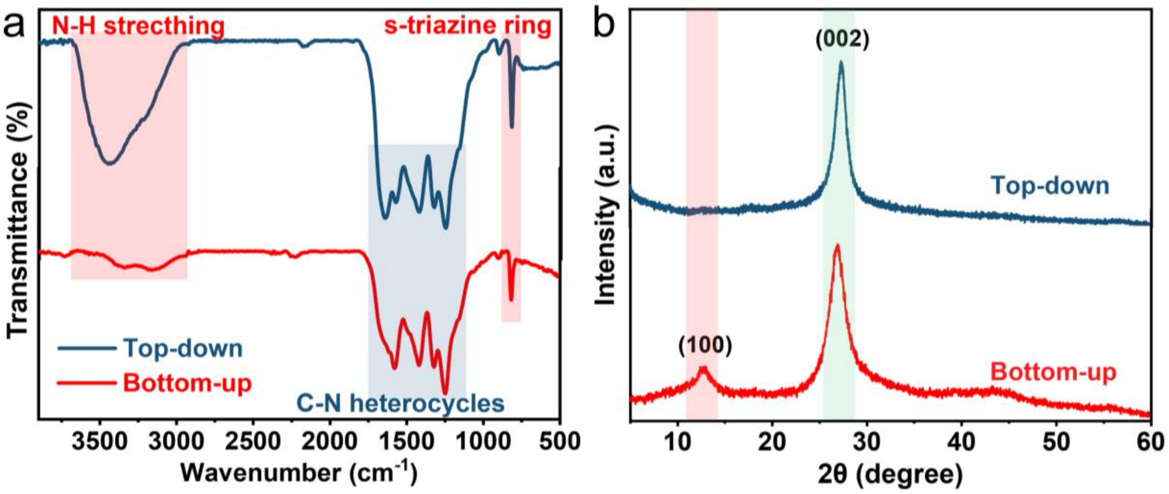

Supplementary Fig. 4. a) FTIR patterns and b) XRD patterns of g-C_3N_4 nanosheets prepared through the top-down and bottom-up method, respectively.

As shown in Supplementary Fig. 4a, the characteristic peak at 810 cm^{-1} is ascribed to the vibrations of the tri-s-triazine ring. The wide bands at 1690-1150 cm^{-1} and 3680-2970 cm^{-1} belong to the stretching vibrations of C-N heterocycles and the N-H stretching vibrations of the terminal NH_2/-NH of g-C_3N_4, respectively[3]. In the XRD patterns (Figure S4b), the (100) peak results from the lattice planes along c-axis due to the 2D planer disorder as shown in Supplementary Fig. 4b[4]. Another reflection was observed at $2\theta = 27.46°$, which is attributed to the staking motif of the very same tri-s-triazine on top of each other, confirming the successful synthesis of g-C_3N_4 nanosheets through this method.

The XRD and FTIR results of the two g-C_3N_4 nanosheets confirmed that the structures of the two types of nanosheets were similar. However, the in-planar atomic structure of g-C_3N_4 was easy to be destroyed during the top-down methods[5,6], which can be confirmed the XRD results that the (100) plane was not detected in the g-C_3N_4 nanosheets prepared through the top-down method.

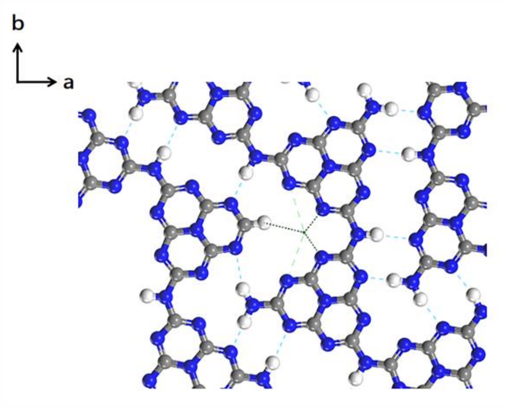

Supplementary Fig. 5. Schematic diagram of the hydrogen bond structure in g-C_3N_4 nanosheets. C: gray; N: blue; H: white.

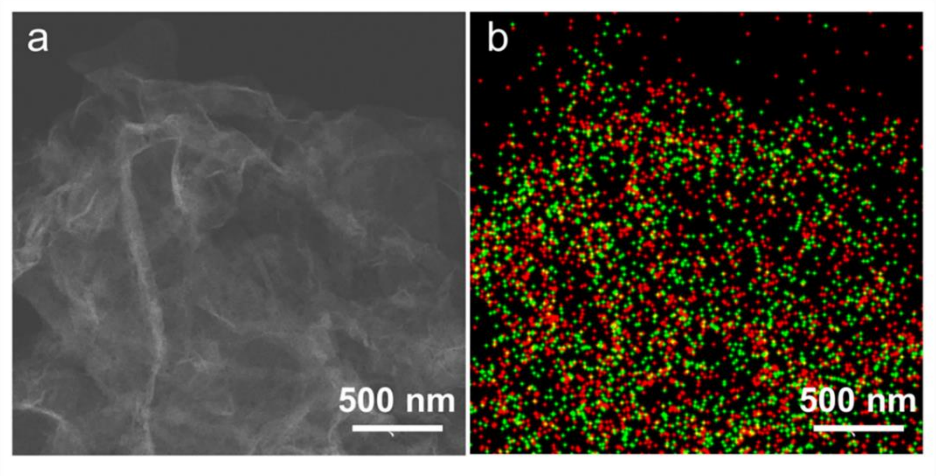

Supplementary Fig. 6. (a) TEM image and (b) corresponding EDXS mapping image of the bottom-up g-C_3N_4 nanosheets. Red for C atoms and green for N atoms.

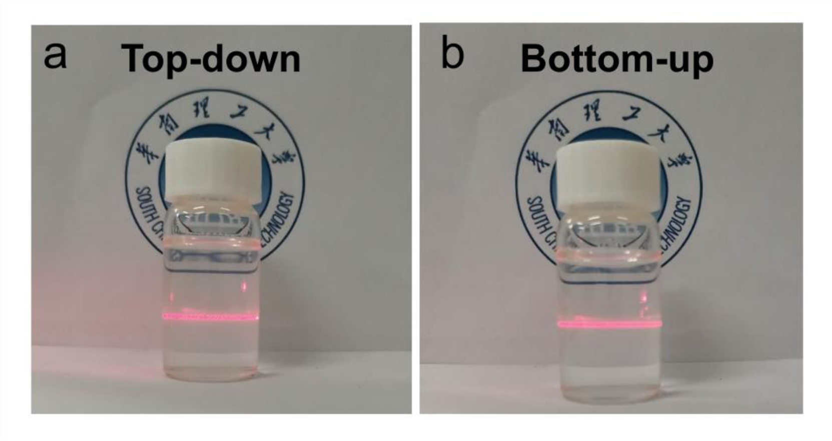

Supplementary Fig. 7. The Tyndall scattering effect in the g-C_3N_4 nanosheets colloidal solution: (a) top-down; (b) bottom-up.

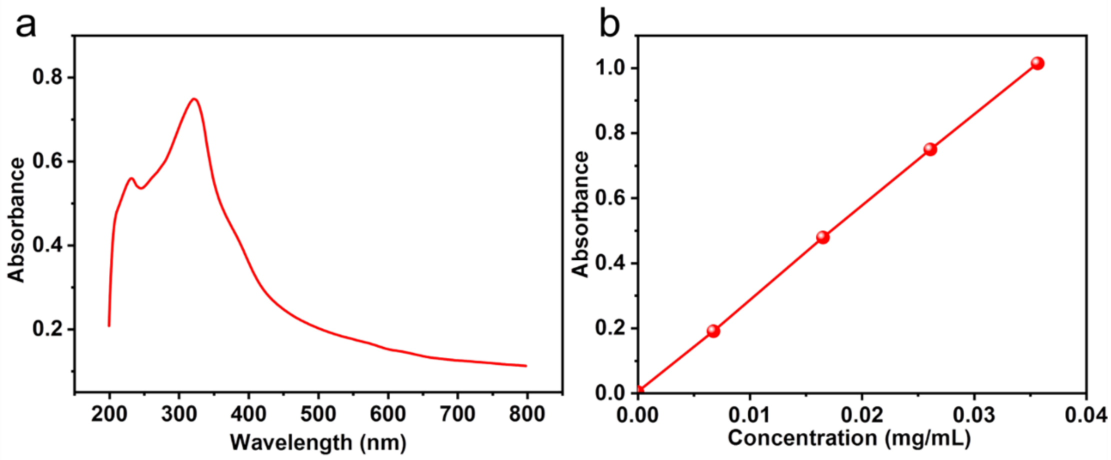

Supplementary Fig. 8. (a) UV-vis absorption spectra of the g-C_3N_4 nanosheets suspension. (b) Dependence of the UV absorbance on the g-C_3N_4 nanosheets suspension concentrations. A strong peak at 322 nm can be observed. Moreover, the absorbance showed a strong linear relationship with the g-C_3N_4 nanosheets concentration.

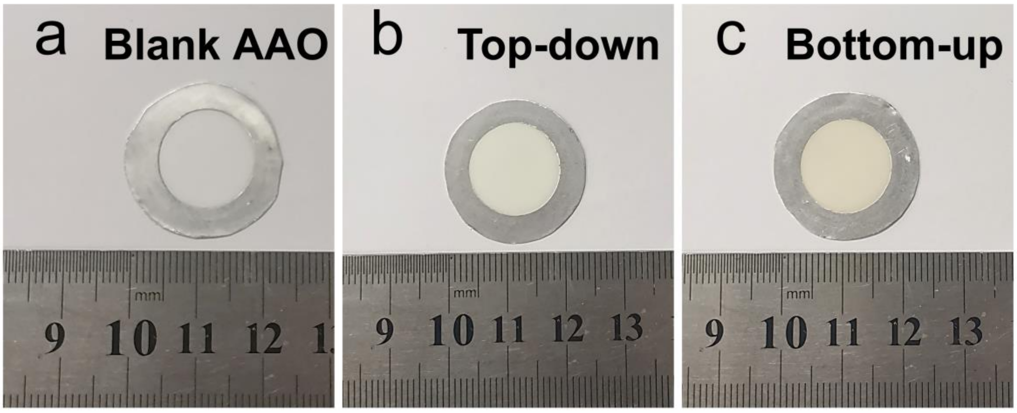

Supplementary Fig. 9. Light yellow g-C_3N_4 membranes supported on AAO were observed after filtration of the g-C_3N_4 suspension. (a) Blank AAO substrate. g-C_3N_4 membranes were assembled by two types of g-C_3N_4 nanosheets prepared through the (b) top-down and (c) bottom-up method, respectively.

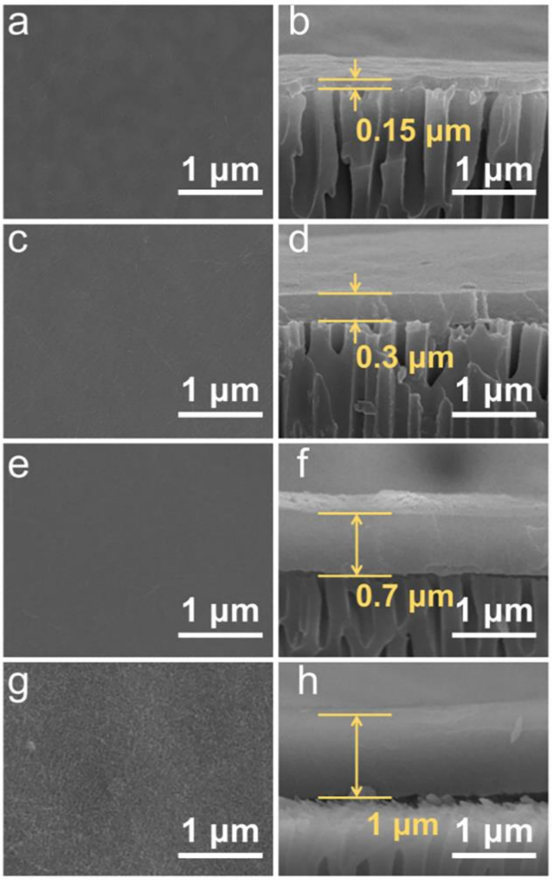

Supplementary Fig. 10. Top-view (a, c, e, g,) and cross-sectional (b, d, f, h,) SEM images of the g-C_3N_4 membranes with different thicknesses from 0.15 μm to 1 μm.

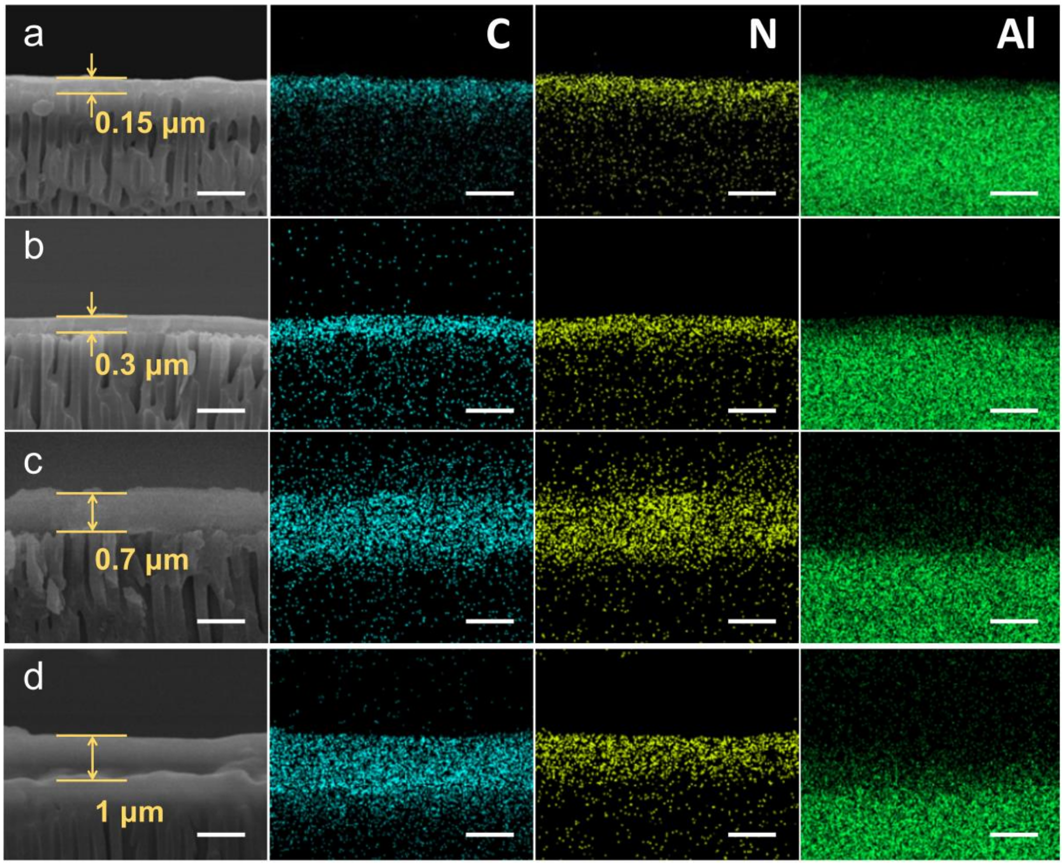

Supplementary Fig. 11. Cross-sectional SEM images and corresponding EDXS mapping images of the g-C_3N_4 membranes with different thicknesses from 0.15 µm to 1 µm. C (blue) and N (yellow) is the tracer for the g-C_3N_4 membranes, and Al (green) is the tracer for the AAO substrate. Scale bar: 1 µm.

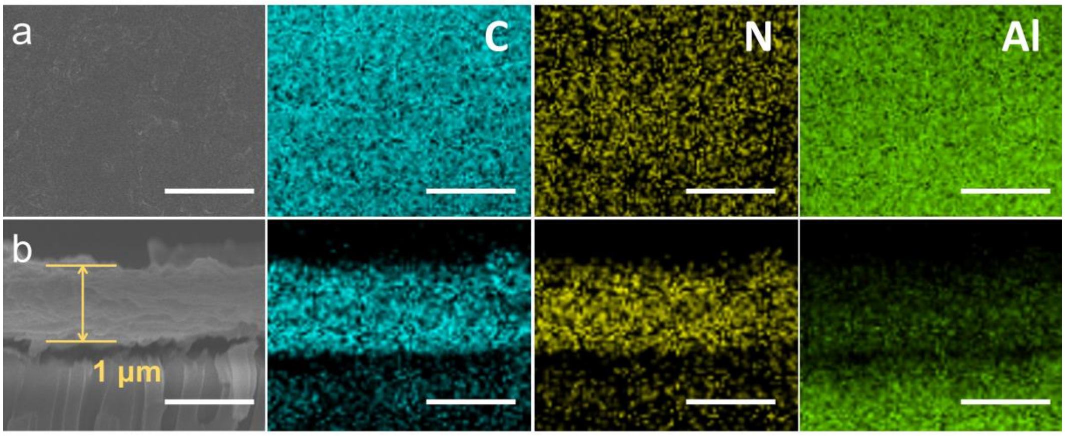

Supplementary Fig. 12. (a) Top-view SEM image and corresponding EDXS mapping images of the g-C_3N_4 membrane assembled by g-C_3N_4 nanosheets prepared through the top-down method. (b) Cross-sectional SEM image and corresponding EDXS mapping images of the g-C_3N_4 membrane assembled by g-C_3N_4 nanosheets prepared through the top-down method. C (blue) and N (yellow) is the tracer for the membrane, and Al (green) is the tracer for the AAO substrate. Scale bar: 1 μm.

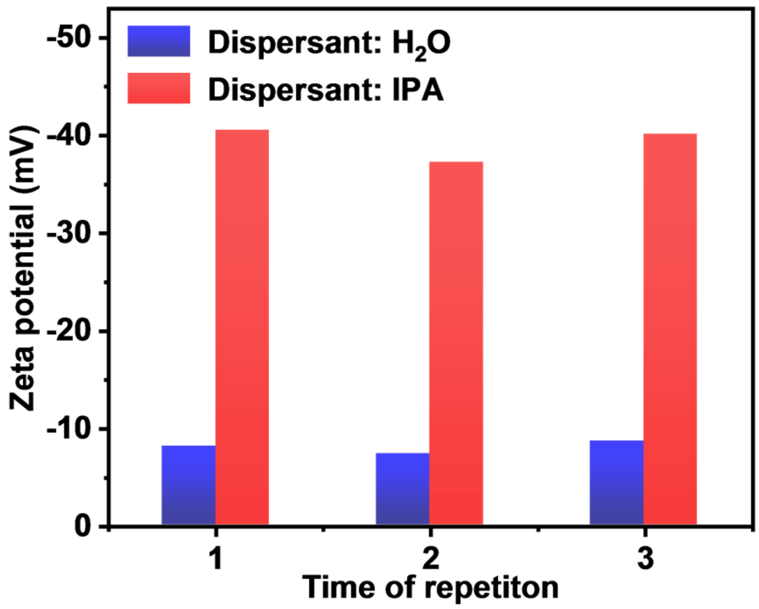

Supplementary Fig. 13. Zeta potential values of g-C_3N_4 nanosheets suspension.

The higher zeta potential of the g-C_3N_4 suspension dispersed in isopropanol compared with those of g-C_3N_4 suspension dispersed in deionized water, demonstrates its more negatively charges, causing the stronger repulsive interactions to weaken the π-π interaction between adjacent nanosheets.

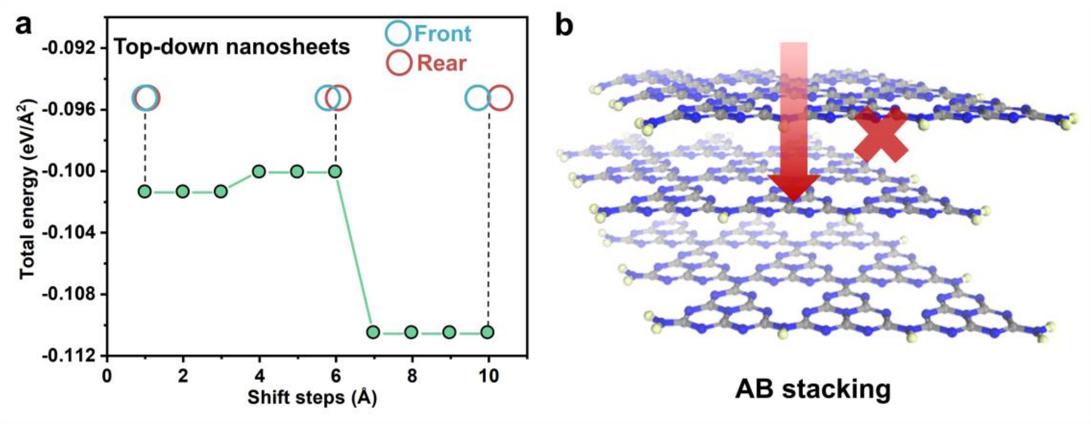

Supplementary Fig. 14. (a) The DFT calculations about stacking states of g-C_3N_4 nanosheets prepared through the top-down method in g-C_3N_4 membranes. The front represents the first layer of nanosheets in a two-layer system of g-C_3N_4 and the rear represents another layer of nanosheets below the first layer of nanosheets. (b) AB stacking of top-down g-C_3N_4 nanosheets. C: gray; N: blue; H: white.

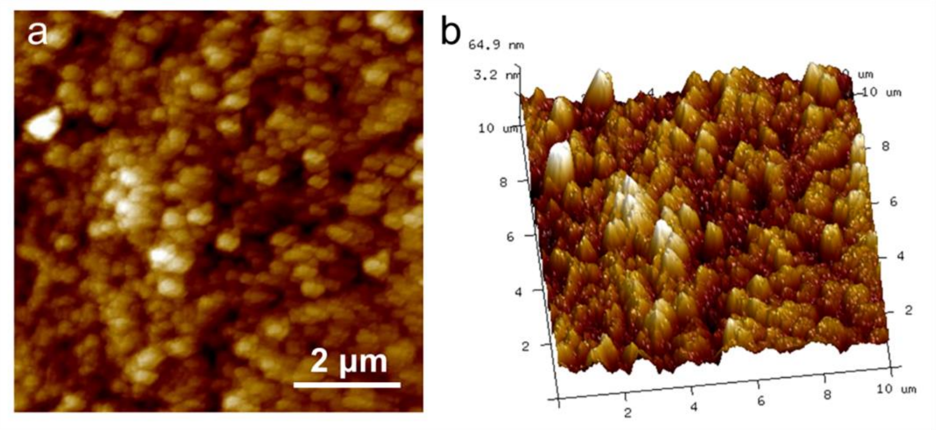

	g-C_3N_4 membrane
**Rq (nm)**	44.8
**Ra (nm)**	36.3

Supplementary Fig. 15. AFM images of the g-C_3N_4 membrane surface. (a) 2D and (b) 3D AFM images.

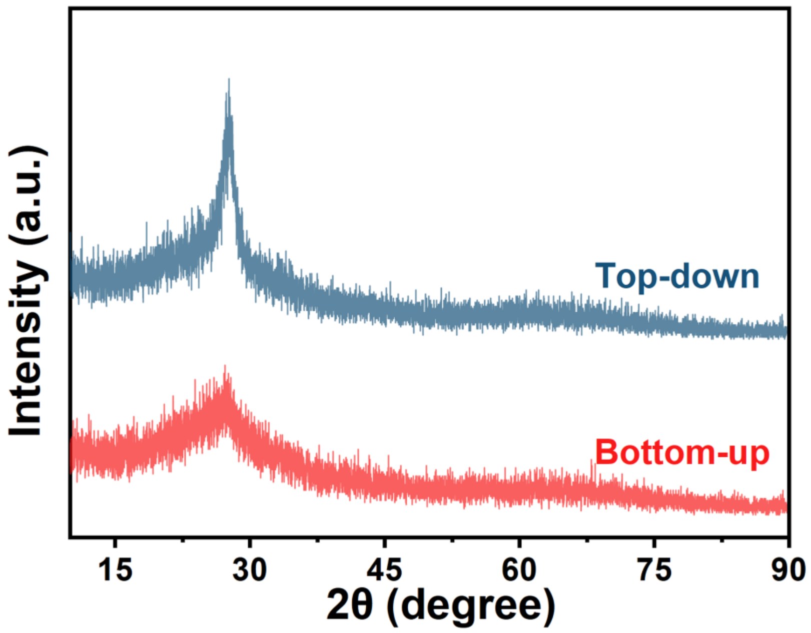

Supplementary Fig. 16. GIAXRD patterns of g-C_3N_4 membranes assembled by two types of g-C_3N_4 nanosheets prepared through the top-down and bottom-up methods, respectively.

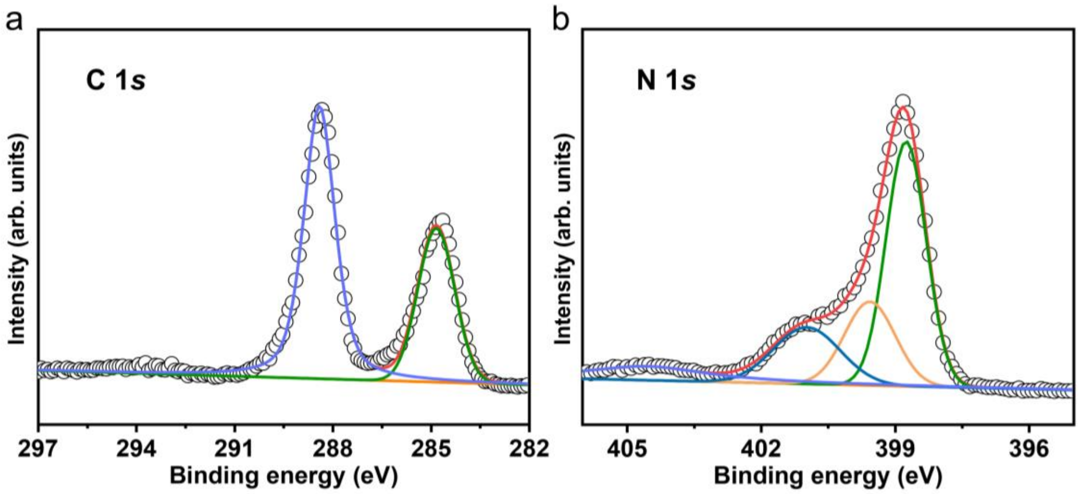

Supplementary Fig. 17. XPS spectra of (a) C 1*s* and (b) N 1*s* core levels of the g-C_3N_4 membrane.

The C 1*s* spectrum of the g-C_3N_4 membrane could be divided into two peaks at 288.4 and 284.8 eV, respectively. The peak at 288.4 eV can be assigned to sp^2 C bonded to N in an aromatic ring, while the peak at 284.8 eV is corresponding to the standard reference carbon. Besides, the N 1*s* spectrum of the g-C_3N_4 membrane can be divided into four peaks at 398.7, 399.6, 400.6 and 404.6 eV. The dominant peak at 398.7 eV is ascribed to the sp^2 nitrogen (C-N=C), and the peaks at approximately 399.6 eV could be attributed to the bridging N atoms in $N(-C)_3$. The peak at 400.6 eV is the terminal amino group (NH_2 or NH). The weak peak at 404.6 eV is ascribed to the positive charge localization in the heterocycles.

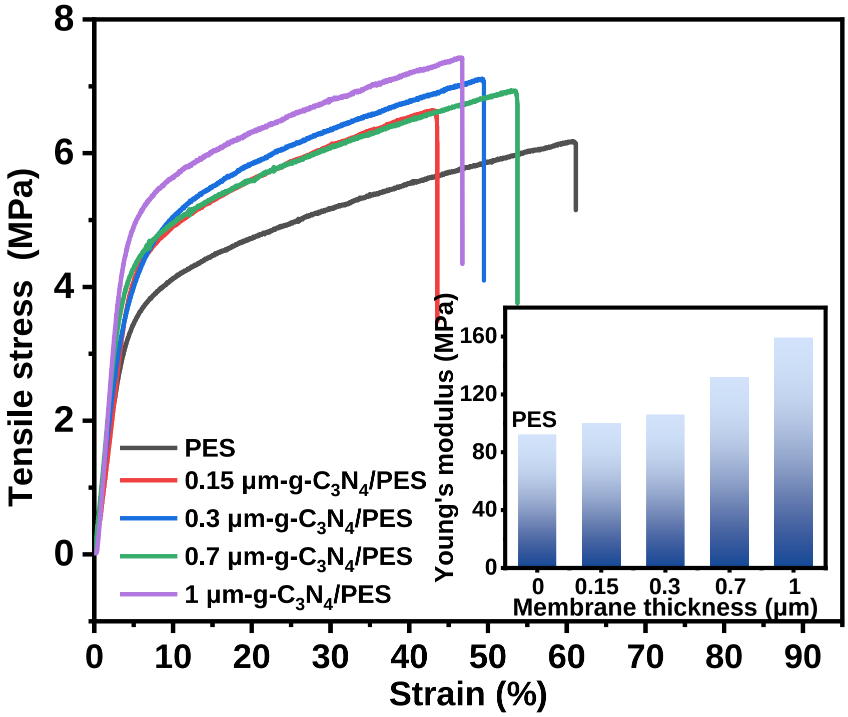

Supplementary Fig. 18. Stress-strain curves of the PES-supported g-C_3N_4 membranes with different thicknesses from 0.15 μm to 1 μm, and the bare PES substrate.

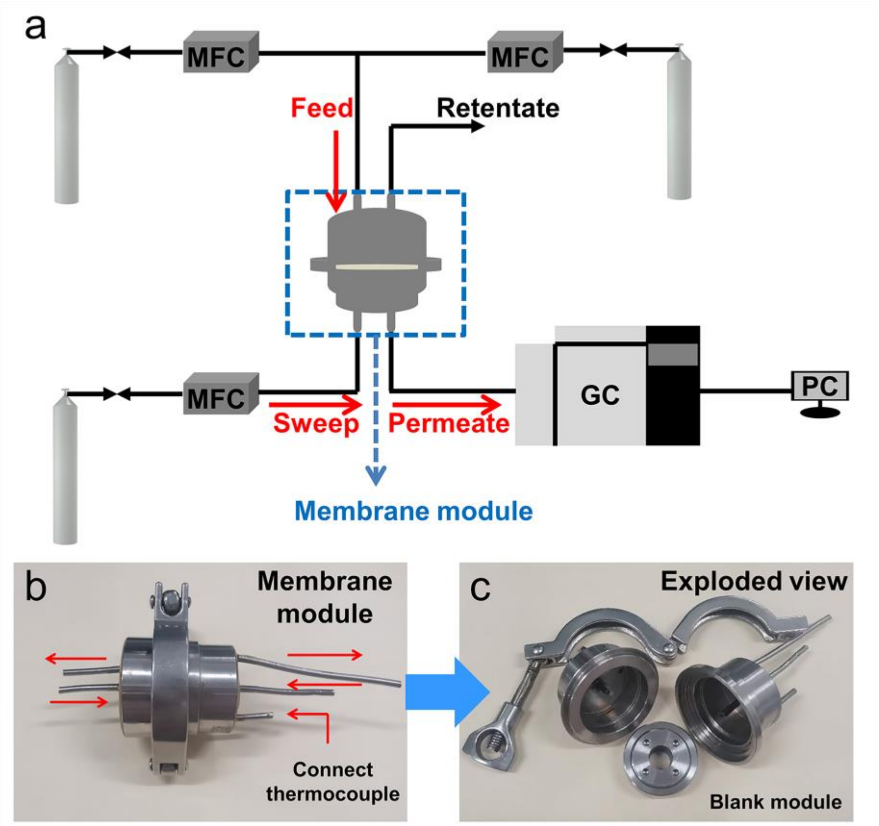

Supplementary Fig. 19. (a) Apparatus scheme of a homemade Wicke-Kallenbach permeation cell for gas separation. MFC: Mass flow controller (Qixinghuachuang, D07-19B). GC: Gas chromatograph (Agilent 7890A) with a thermal conductivity detector (TCD) and a flame ionization detector (FID). Photos of the membrane module for gas permeation. (b) Overview photo of the membrane module. (c) Inside view photo of the membrane module.

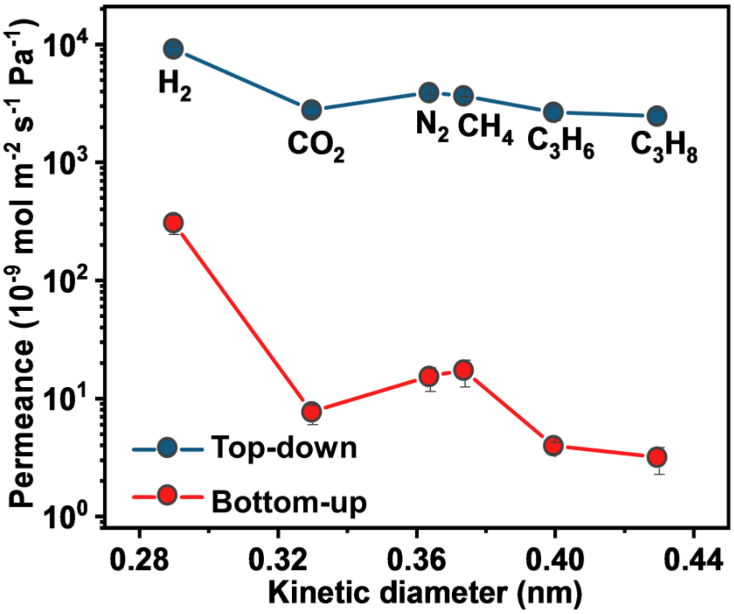

Supplementary Fig. 20. Single gas permeance through the g-C_3N_4 membranes assembled by two types of g-C_3N_4 nanosheets prepared through the top-down and bottom-up method, respectively, which have the same thickness (1 μm) at room temperature and 1 bar. Errors bars indicate the standard deviation of three measurements.

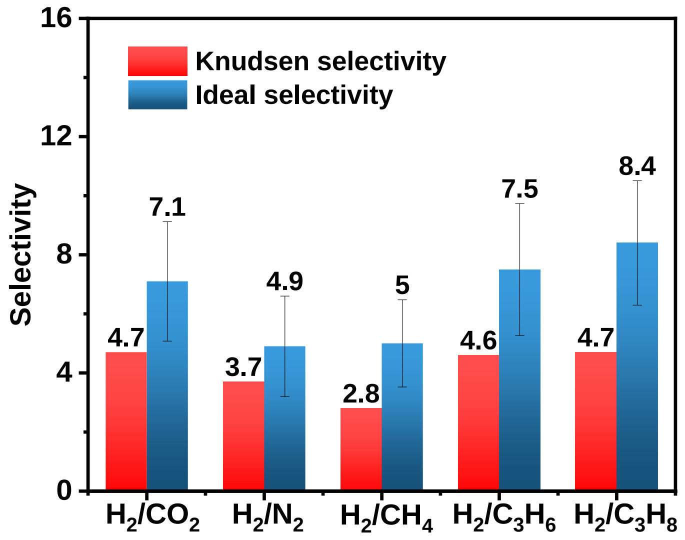

Supplementary Fig. 21. The gas selectivity of a 1-μm-thick g-C_3N_4 membrane assembled by the g-C_3N_4 nanosheets prepared through the top-down method, at room temperature and 1 bar. Errors bars indicate the standard deviation of three measurements.

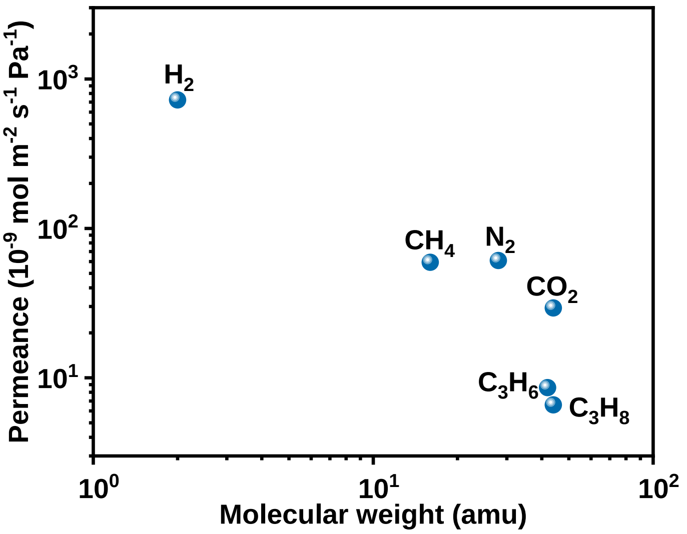

Supplementary Fig. 22. Single gas permeance through a 1-μm-thick g-C_3N_4 membrane as a function of gas molecular weight (amu, atomic mass unit) at room temperature and 1 bar. There is no obvious proportional relationship between the gas permeances and gas molecular weight.

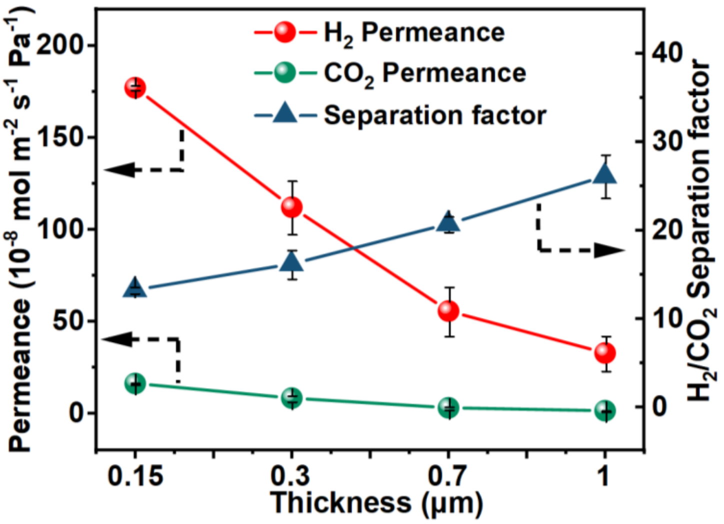

Supplementary Fig. 23. Permeance and separation factors of the 1-μm thick g-C_3N_4 membrane in the equimolar mixed-gas permeation. Errors bars indicate the standard deviation of three measurements.

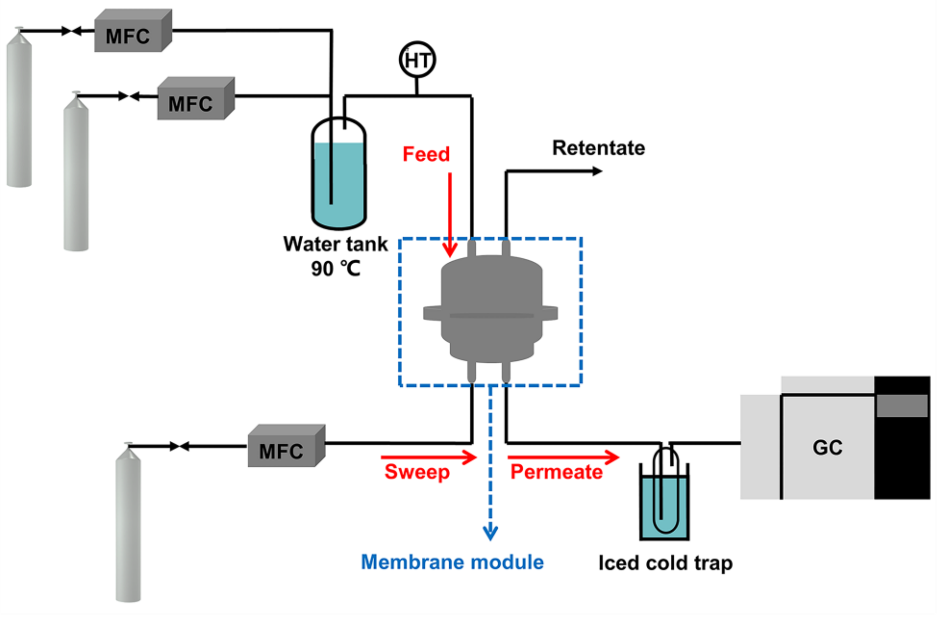

Supplementary Fig. 24. Illustration of the gas permeation rig with humidity control. MFC: Mass flow controller. GC: Gas chromatograph. HT: Humidity transmitter.

Supplementary Note 1

$$a_w = \frac{\mathrm{RH}}{100} = \frac{P_{H_2O}}{P_{sat}} \quad (1)$$

Table. Saturated vapor pressure of water at different temperatures

Temperature	Saturated vapor pressure of H_2O
25 °C	3.169×10^3 Pa
90 °C	70.12×10^3 Pa
120 °C	198.48×10^3 Pa

When the membrane was tested at room temperature with controlling the temperature of the water tank at 25 °C, water vapor with a partial pressure of 3.169×10^3 Pa is introduced into the feed gas. As a result, in a gas stream at 25 °C and 1 atm (Pressure: 101.32×10^3 Pa), the water activity (a_w) is 0.03.

When the membrane was tested at 120 °C with controlling the temperature of the water tank at 90 °C, water vapor with a partial pressure of 7.012×10^4 Pa is introduced into the feed gas. As a result, in a gas stream at 120 °C and 1 atm (Saturated vapor pressure: 1.9848×10^5 Pa), the water activity (a_w) is 0.353.

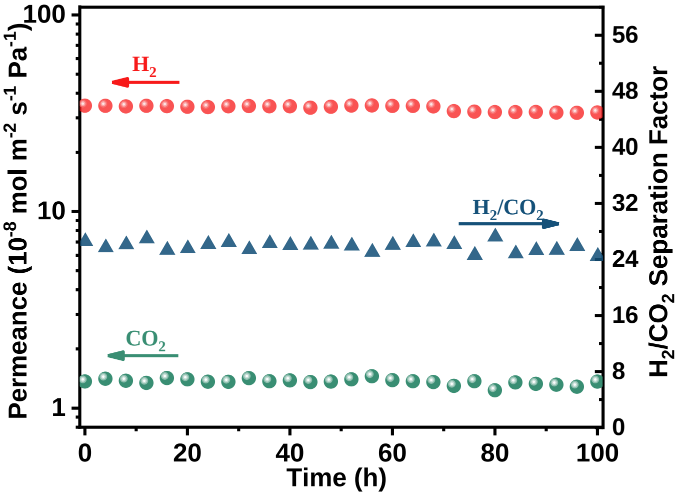

Supplementary Fig. 25. Long-term stability of a 1-μm-thick g-C_3N_4 membrane in the equimolar mixed-gas permeation with an added 3 vol% water vapor at room temperature and 1 bar.

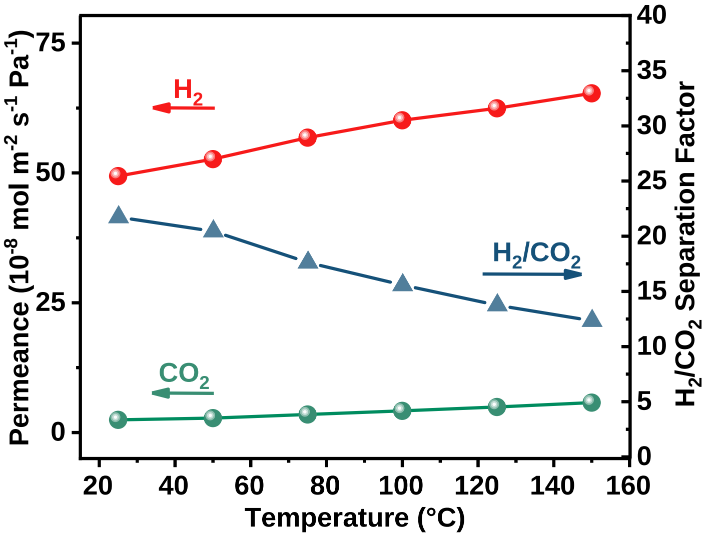

Supplementary Fig. 26. Gas permeances and H_2/CO_2 separation factor of a 1-µm-thick g-C_3N_4 membrane as a function of temperature in the equimolar mixed-gas permeation and 1 bar.

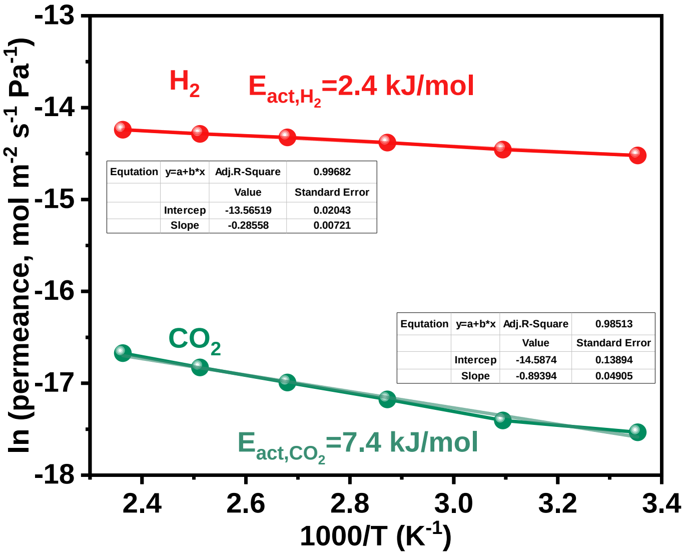

Supplementary Fig. 27. Arrhenius temperature dependence of H_2 and CO_2 permeances through the g-C_3N_4 membrane at room temperature with equimolar mixed gas feeding.

The Arrhenius equation states the temperature dependence of gas permeation:

$$P = A\exp\left(-\frac{E_{\mathrm{act}}}{\mathrm{R}T}\right) \quad (2)$$

$$\ln P = -\frac{E_{\mathrm{act}}}{\mathrm{R}} \cdot \frac{1}{T} + \mathrm{C} \quad (3)$$

where P is the gas permeance, A is the pre-exponential factor, E_{act} is the apparent activation energy, R is the ideal gas constant (8.314 J mol^{-1} K^{-1}), and T is the absolute Kelvin temperature (K). ln(P) versus 1/T displays a straight line in which the slope was used to calculate E_{act}.

As shown in Supplementary Fig. 27, the $E_{\mathrm{act,H_2}}$ is about 2.4 kJ mol^{-1}, and $E_{\mathrm{act,CO_2}}$ is about 7.4 kJ mol^{-1}. The apparent activation energy is an association between diffusion activation energy and adsorption heat.

$$E_{\mathrm{act}} = E_{\mathrm{diff}} - \Delta H_{\mathrm{ads}} \quad (4)$$

CO_2 adsorption on g-C_3N_4 should release more heat than H_2, considering the weak adsorption of H_2 on g-C_3N_4. As a result, when diffusion through the g-C_3N_4 membrane, CO_2 should also have a higher activation energy than H_2. It is, therefore, inferred that CO_2 diffusing through g-

C_3N_4 membranes was a much more activated process than H_2, and it was a tighter fit for CO_2 in g-C_3N_4 flakes in high temperatures. The theory also explains why the H_2/CO_2 separation factor decreased with an increasing temperature. Because CO_2 permeance rose faster with temperature than H_2, CO_2 diffusion was more activated than H_2 in the g-C_3N_4 membrane.

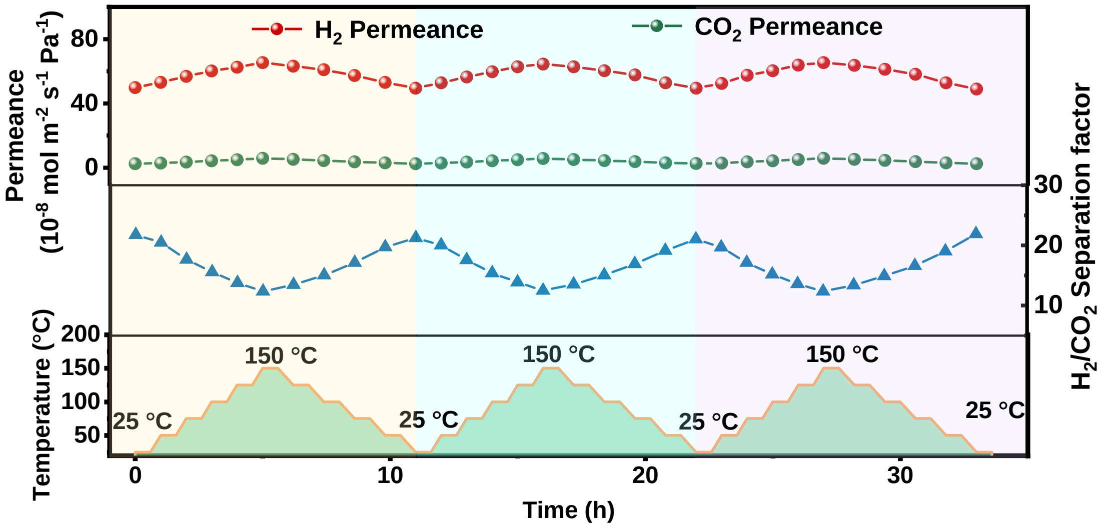

Supplementary Fig. 28. Three temperature cycles operation for a 1-μm-thick bottom-up g-C_3N_4 membrane with equimolar H_2/CO_2 mixture and 1 bar. The permeances of the bottom-up g-C_3N_4 membrane in the temperature-swing fluctuate slightly, however, the separation factor all return to the original level.

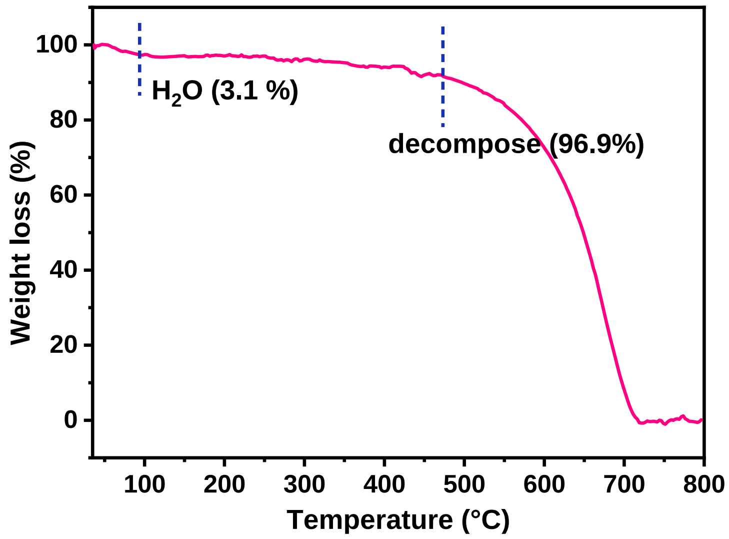

Supplementary Fig. 29. Thermogravimetric analysis result of the g-C_3N_4 nanosheets. The curve shows a relatively good thermal stability of g-C_3N_4 nanosheets from room temperature to 480 °C in a nitrogen atmosphere with a heating rate of 10 °C min^{-1}. However, there was a slight weight loss (3.1%) of the g-C_3N_4 nanosheets during the heating process. The adsorbed H_2O might cause the weight loss to occur at ~100 °C.

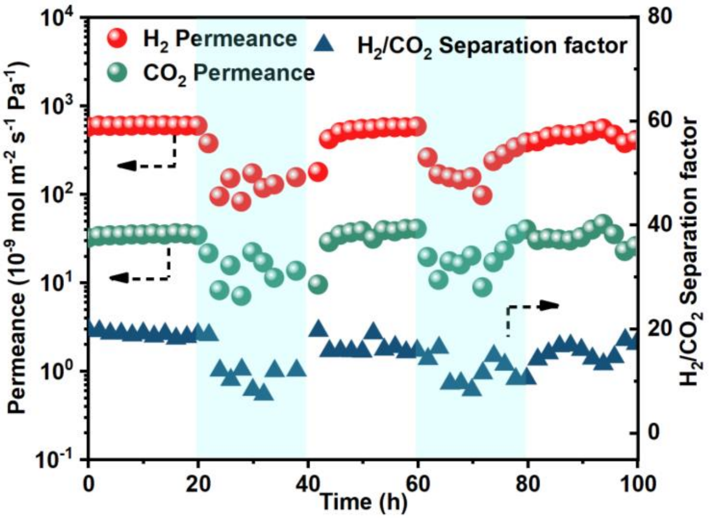

Supplementary Fig. 30. Long-term stability for H_2/CO_2 separation under dry and wet gas mixture (water activity of 0.353, marked by blue areas) at 120 °C and 1 bar.

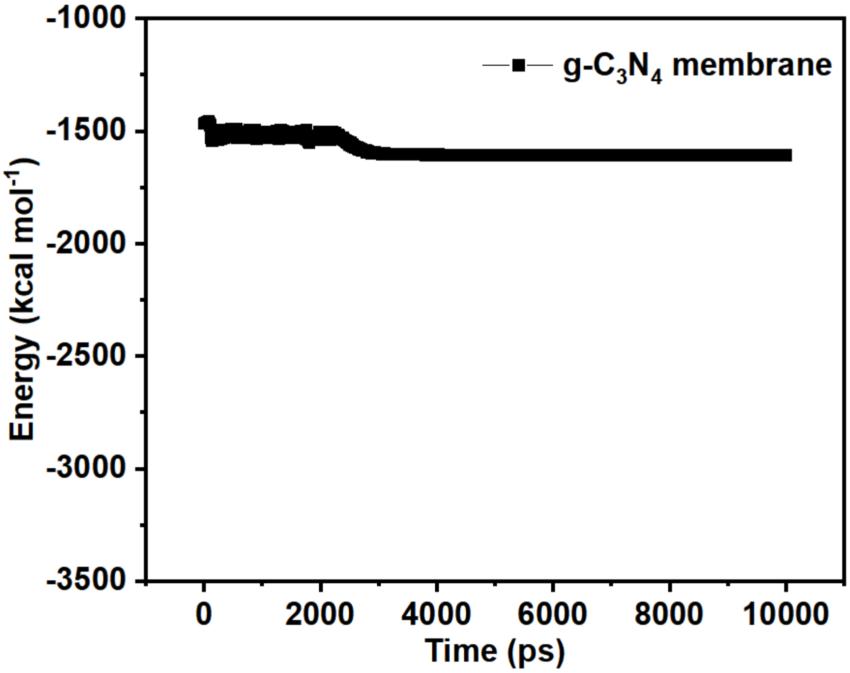

Supplementary Fig. 31. Total energy for g-C_3N_4 membranes during the MD simulations.

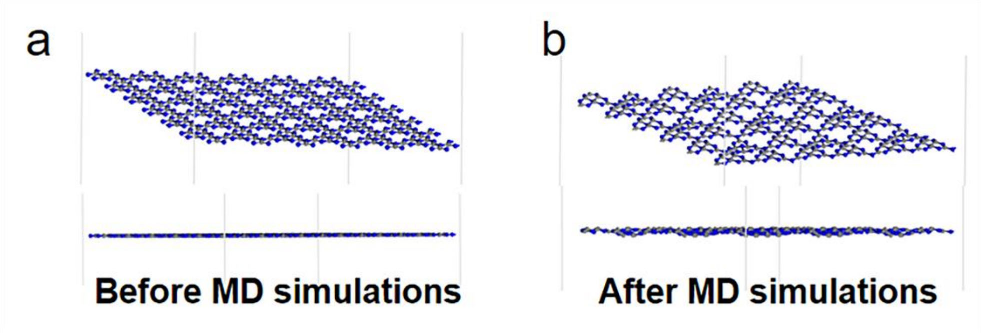

Supplementary Fig. 32. The schematic diagram of the g-C_3N_4 layer before and after the MD simulations. C: gray; N: blue.

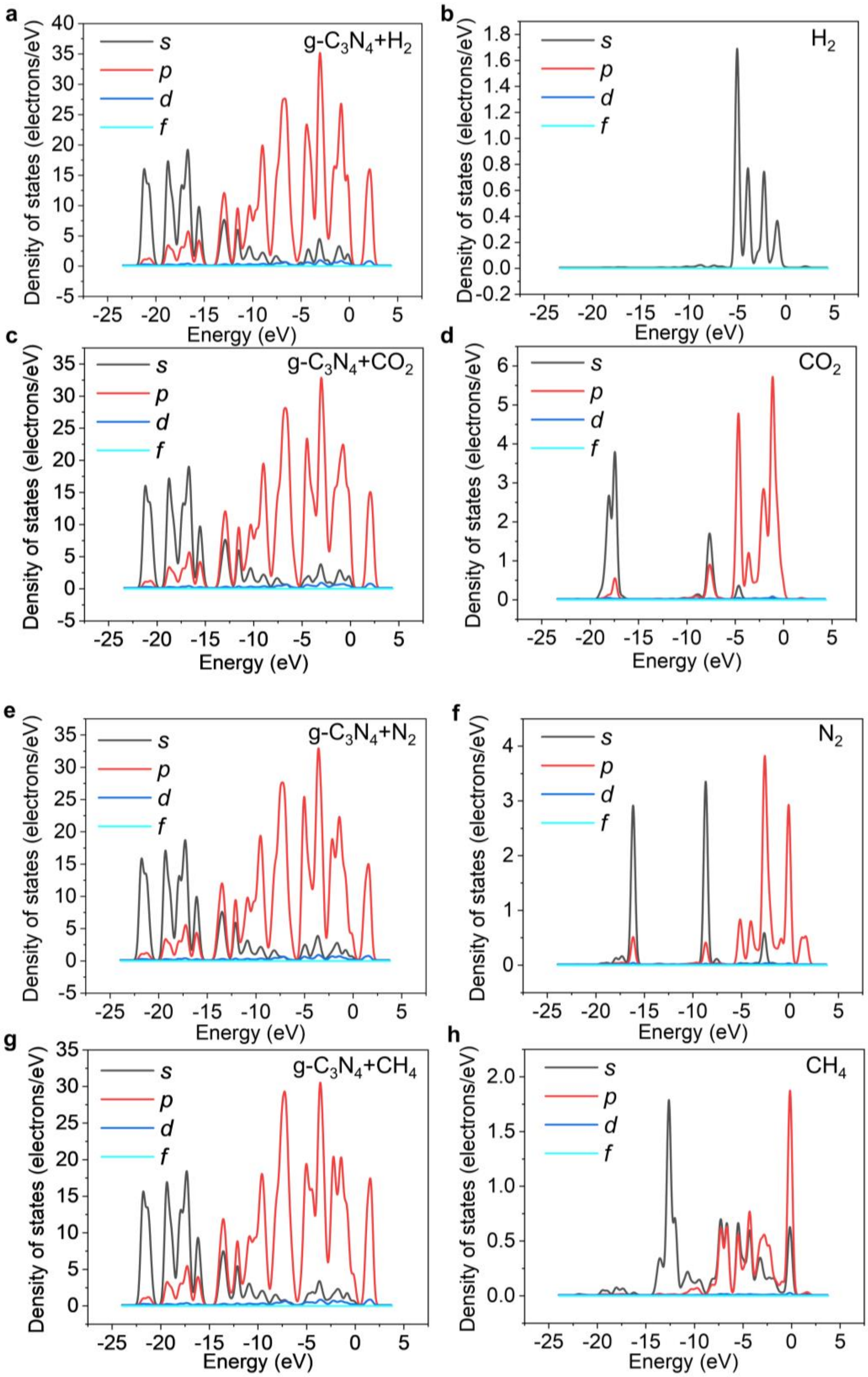

Supplementary Fig. 33. (a, c, e, g) PDOS of the g-C_3N_4 adsorbed by gas molecules (H_2, CO_2, N_2, CH_4). (b, d, f, h) PDOS of H_2, CO_2, N_2, CH_4 gas molecules models. The assignment of color: black = *s* orbit; red = *p* orbit; blue = *d* orbit, cyan = *f* orbit.

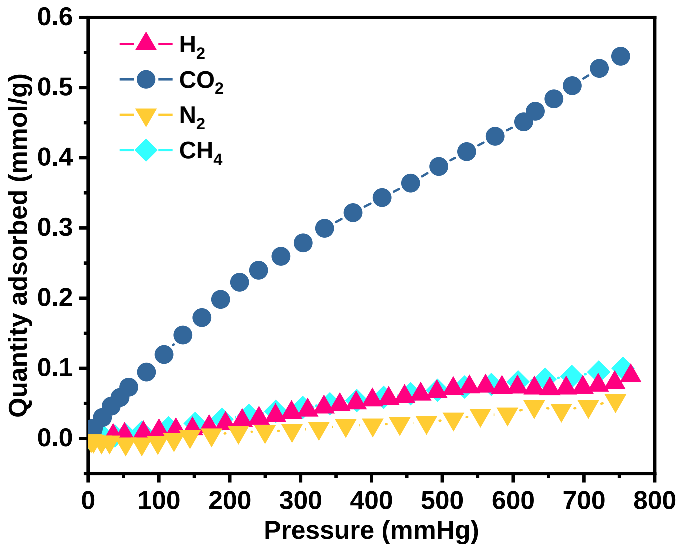

Supplementary Fig. 34. Adsorption isotherms of H_2, CO_2, N_2, and CH_4, on g-C_3N_4 nanosheets at room temperature.

2. Supplementary Tables

Supplementary Table 1. Organic elemental analysis of g-C_3N_4 nanosheets prepared through the top-down and bottom-up methods.

Sample	C (wt %)	N (wt %)	H (wt %)	C/N (atom ratio)
**Top-down**	28.00	50.32	2.705	0.649
**Bottom-up**	32.93	56.41	2.100	0.681

Supplementary Table 2. Mechanical properties of the PES-supported g-C_3N_4 membranes and the bare PES substrate.

**Membrane**	**Thickness (μm)**	**Strain (%)**	**Tensile stress (MPa)**	**Young's modulus (MPa)**
PES	0	60.86	6.18	92.29
g-C_3N_4/PES	0.15	43.06	6.64	99.9
g-C_3N_4/PES	0.3	49.28	7.11	106.09
g-C_3N_4/PES	0.7	52.98	6.94	131.83
g-C_3N_4/PES	1.0	46.5	7.43	158.95

Supplementary Table 3. Separation performance of 1-μm-thick g-C_3N_4 membranes assembled by two types of g-C_3N_4 nanosheets prepared through the top-down and bottom-up method in single gas permeation experiments.

**Name**	**Membrane**	**Performance of g-C_3N_4 membrane**										
								**Ideal selectivity**				
		**H_2**	**CO_2**	**N_2**	**CH_4**	**C_3H_6**	**C_3H_8**	**H_2/CO_2**	**H_2/N_2**	**H_2/CH_4**	**H_2/C_3H_6**	**H_2/C_3H_8**
**Top-down**	M1	4.74×10^{-6}	1.30×10^{-6}	1.96×10^{-6}	1.79×10^{-6}	1.22×10^{-6}	1.16×10^{-6}	9.83	5.60	6.32	10.68	11.35
	M2	3.58×10^{-6}	1.24×10^{-6}	1.98×10^{-6}	1.80×10^{-6}	1.09×10^{-6}	9.68×10^{-7}	5.01	2.62	2.98	5.91	6.81
	M3	4.65×10^{-6}	1.79×10^{-6}	1.72×10^{-6}	1.87×10^{-6}	1.88×10^{-6}	1.68×10^{-6}	6.44	6.63	5.84	5.98	6.97
	Average	4.32×10^{-6}	1.44×10^{-6}	1.89×10^{-6}	1.82×10^{-6}	1.39×10^{-6}	1.27×10^{-6}	7.09	4.95	5.05	7.52	8.38
	Standard deviation	5.27×10^{-7}	2.45×10^{-7}	1.17×10^{-7}	3.55×10^{-8}	3.47×10^{-7}	3.00×10^{-7}	2.02	1.70	1.48	2.23	2.11
**Bottom-up**	M4	2.63×10^{-7}	6.92×10^{-9}	1.23×10^{-8}	1.33×10^{-8}	3.45×10^{-9}	2.53×10^{-9}	41.12	23.00	21.34	82.51	112.61
	M5	3.81×10^{-7}	9.28×10^{-9}	1.96×10^{-8}	2.28×10^{-8}	4.39×10^{-9}	4.16×10^{-9}	45.77	21.65	18.55	96.86	102.21
	M6	2.60×10^{-7}	5.99×10^{-9}	1.25×10^{-8}	1.42×10^{-8}	3.63×10^{-9}	2.48×10^{-9}	46.72	22.27	19.70	77.23	113.22
	Average	3.01×10^{-7}	7.40×10^{-9}	1.48×10^{-8}	1.68×10^{-8}	3.82×10^{-9}	3.05×10^{-9}	44.54	22.31	19.86	85.53	109.34
	Standard deviation	5.62×10^{-8}	1.39×10^{-9}	3.36×10^{-9}	4.29×10^{-9}	4.09×10^{-10}	7.83×10^{-10}	2.45	0.55	1.14	8.29	5.05

Supplementary Table 4. H_2/CO_2 separation performance of the g-C_3N_4 nanosheets membranes with different thicknesses from 0.15 μm to 1 μm for equimolar mixed gas feeding.

**Thickness**	**Membrane**	**H_2 Permeance (mol m^{-2} s^{-1} Pa^{-1})**	**CO_2 Permeance (mol m^{-2} s^{-1} Pa^{-1})**	**H_2/CO_2 separation factor**
**0.15 μm**	M1	1.79×10^{-6}	1.63×10^{-7}	13.23
	M2	1.77×10^{-6}	1.66×10^{-7}	12.80
	M3	1.76×10^{-6}	1.54×10^{-7}	13.65
	Average	1.77×10^{-6}	1.61×10^{-7}	13.23
	Standard deviation	1.29×10^{-8}	4.73×10^{-9}	0.35
**0.3 μm**	M4	9.50×10^{-7}	5.76×10^{-8}	18.20
	M5	1.17×10^{-6}	9.34×10^{-8}	14.24
	M6	1.30×10^{-6}	9.39×10^{-8}	16.04
	Average	1.12×10^{-6}	8.19×10^{-8}	16.16
	Standard deviation	1.45×10^{-7}	1.65×10^{-8}	1.61
**0.7 μm**	M7	7.39×10^{-7}	3.98×10^{-8}	20.35
	M8	5.02×10^{-7}	2.71×10^{-8}	19.79
	M9	4.25×10^{-7}	2.05×10^{-8}	21.90
	Average	5.55×10^{-7}	2.91×10^{-8}	20.68
	Standard deviation	1.34×10^{-7}	8.01×10^{-9}	0.89
**1 μm**	M10	1.95×10^{-7}	8.51×10^{-9}	23.50
	M11	4.16×10^{-7}	1.50×10^{-8}	29.36
	M12	3.71×10^{-7}	1.58×10^{-8}	25.36
	Average	3.27×10^{-7}	1.31×10^{-8}	26.07
	Standard deviation	9.52×10^{-8}	3.26×10^{-9}	2.44

Supplementary Table 5. Summary of the H_2/CO_2 separation performance of membranes (data in main text Figure 3d).

Membrane Material		Thickness (μm)	Temperature (°C)	H_2 Permeance (mol m^{-2} s^{-1} Pa^{-1})	Separation factor (H_2/CO_2)	Reference
**CMS**	CMS	--	25	5.33×10^{-9}	15	7
	CMS	10.69	--	5.61×10^{-8}	2.5	8
**PIM**	PIM-EA-TB	181	25	1.40×10^{-8}	1.09	9
	PIM-1-450	33	35	2.02×10^{-9}	8	10
**PBI**	Pure PBI	~50	35	1.93×10^{-11}	7.1	11
	Pure PBI	~2	35	6.00×10^{-10}	8.6	12
**GO**	GO	0.009	20	1.15×10^{-7}	3400	13
	GO	0.02	20	3.40×10^{-7}	240	14
	ZIF-8/GO	~0.02	25	8.00×10^{-8}	406	15
	EFDA-GO	1	25	2.80×10^{-7}	33	16
	SOD/GO-M1	1.1~1.2	25	3.50×10^{-7}	~105	17
	HGO-4h@2	1.3	35	4.87×10^{-9}	~20	18
**MoS_2**	1T MoS_2	1	25	4.43×10^{-7}	7.6	19
	2H MoS_2	1	25	5.80×10^{-7}	6	19
	MoS_2	0.06	35	8.16×10^{-7}	4.4	20
**MOFs**	ZIF-8	4.2	25	1.34×10^{-7}	~2400	21
	ZIF-8	6	30	1.57×10^{-7}	4.6	22
	ZIF-8	12	25	9.82×10^{-8}	6	23
	ZIF-8	2	25	4.22×10^{-7}	3.28	24
	ZIF-22	40	50	1.63×10^{-7}	7.2	25
	HKUST-1	60	25	9.82×10^{-7}	6.8	26
	CAU-1	4	25	1.07×10^{-7}	12.3	27
	SIM-1	25	30	8.16×10^{-8}	2.3	28
	Amine-Mg-MOF-74	10	25	7.47×10^{-8}	28	29
	MIL-96 (Al)	8	25	5.11×10^{-7}	8.8	30
	ZIF-8/g-C_3N_4	0.24	25	6.70×10^{-8}	42	31
	COF-MOF	97.2	25	3.77×10^{-7}	13.5	32
	2D ZIFs	--	25	9.00×10^{-7}	291	33
	2D MOFs	0.05	30	2.00×10^{-7}	53	34
	2D MOFs	0.04	20	2.38×10^{-7}	245	35
	2D MOFs	0.44	25	9.50×10^{-7}	13	36
**COFs**	2D COFs	2	25	1.22×10^{-6}	31.6	37
	COF-LZU1–ACOF-1	1	25	2.20×10^{-7}	24.2	38
	[COF-300]-[UiO-66]	100	25	3.91×10^{-7}	17.2	39
**Mxene**	MXene	2	25	3.71×10^{-7}	167	40
**g-C_3N_4**		0.15	25	1.77×10^{-6}	13.2	This work
		0.3	25	1.30×10^{-6}	16	This work

Supplementary Table 6. The transition state energy E_{TS} (eV), stable state energy E_{SS} (eV) and energy barriers E_b (eV) of gas molecules on the g-C_3N_4 lattice.

	Transition state		Stable state		E_b (eV)
	Height (Å)	E_{TS} (eV)	Height (Å)	E_{SS} (eV)	
H_2	0	0.0346	1	-0.0976	0.1322
CO_2	0	0.7691	3	-0.1996	0.9687
N_2	0	0.5485	2	-0.2339	0.7824
CH_4	0	0.5458	1	-0.2447	0.7905

Supplementary Note 2

The theoretical H_2/gas molecules selectivity can be calculated from the DFT data based on the Arrhenius equation defined in equation (5):

$$S_{\mathrm{H_2/gas}} = \frac{r_{\mathrm{H_2}}}{r_{\mathrm{gas}}} = \frac{A_{\mathrm{H_2}}\exp(-E_{\mathrm{H_2}}/k_{\mathrm{B}}T)}{A_{\mathrm{gas}}\exp(-E_{\mathrm{gas}}/k_{\mathrm{B}}T)} \quad (5)$$

where r is the rate of translocation and A is the ideal gas diffusion prefactor. $E_{\mathrm{H_2}}$ and E_{gas} are the diffusion energy barrier for H_2 and other gas molecules, respectively; k_B is the Boltzmann constant and T is the temperature. Assuming that $A_{\mathrm{H_2}}$ and A_{gas} are within the same order of magnitude, an approximate gas pair selectivity can be calculated. For example: H_2/CO_2 selectivity is calculated as:

$$S_{\mathrm{H_2/CO_2}} = \frac{A_{\mathrm{H_2}}\exp(-E_{\mathrm{H_2}}/k_{\mathrm{B}}T)}{A_{\mathrm{CO_2}}\exp(-E_{\mathrm{CO_2}}/k_{\mathrm{B}}T)}$$

$$= \frac{\exp[-0.1322 \times 1.6 \times 10^{-19}/(1.3806505 \times 10^{-23} \times 298)]}{\exp[-0.9687 \times 1.6 \times 10^{-19}/(1.3806505 \times 10^{-23} \times 298)]} = 1.3 \times 10^{14}$$

Consequently, the DFT calculations predicted an excellent selectivity of H_2/CO_2 with 1.3×10^{14} at room temperature.

Supplementary References

1. Wang, R. D., Xu, B. B., Wang, J. C., Wang, X. L. & Yao, Y. F. Selective hydrogen–deuterium exchange in graphitic carbon nitrides: probing the active sites for photocatalytic water splitting by solid-state NMR. *J. Mater. Chem. A.* **9**, 3985-3994 (2021).

2. Hu, Y. C. et al. Synthesis of ^{13}C-,^{15}N-Labeled graphitic carbon nitrides and NMR-Based evidence of hydrogen-bonding assisted two-dimensional assembly. *Chem. Mater.* **29**, 5080-5089 (2017).

3. Cao, K. T. et al. Highly water-selective hybrid membrane by incorporating g-C_3N_4 nanosheets into polymer matrix. *J. Membr. Sci.* **490**, 72-83 (2015).

4. Lotsch, B. V. et al. Unmasking melon by a complementary approach employing electron diffraction, solid-state NMR spectroscopy, and theoretical calculations-structural characterization of a carbon nitride polymer. *Chem. Eur. J.* **13**, 4969-4980 (2007).

5. Dong, X. P. & Cheng, F. X. Recent development in exfoliated two-dimensional g-C_3N_4 nanosheets for photocatalytic applications. *J. Mater. Chem. A* **3**, 23642-23652 (2015).

6. Wang, Y. J. et al. Water transport with ultralow friction through partially exfoliated g-C_3N_4 nanosheet membranes with self-supporting spacers. *Angew. Chem. Int. Ed.* **56**, 8974-8980 (2017).

7. Maheswari, A. U. & Palanivelu, K. Carbon dioxide capture by facilitated transport membranes: a review. *Int. J. Glob. Warm.* **12**, 1-49 (2017).

8. Li, J. Y., Cheng, P. Y., Lin, M. D., Wey, M. Y. & Tseng, H. H. Uniformity control and ultra-micropore development of tubular carbon membrane for light gas separation. *AIChE J.* **66**, 6 (2020).

9. Carta, M. et al. An efficient polymer molecular sieve for membrane gas separations.

Science **339**, 303-307 (2013).

10. He, S. S., Jiang, X., Li, S. W., Ran, F. T., Long, J. & Shao, L. Intermediate thermal manipulation of polymers of intrinsic microporous (PIMs) membranes for gas separations. *AIChE J.* **66**, 10 (2020).

11. Yang, T. X., Xiao, Y. C. & Chung, T. S. Poly-/metal-benzimidazole nano-composite membranes for hydrogen purification. *Energy Environ. Sci.* **4**, 4171-4180 (2011).

12. Yang, T. X., Shi, G. M. & Chung, T. S. Symmetric and asymmetric zeolitic imidazolate frameworks (ZIFs)/polybenzimidazole (PBI) nanocomposite membranes for hydrogen purification at high tmperatures. *Adv. Energy Mater.* **2**, 1358-1367 (2012).

13. Li, H. et al. Ultrathin, Molecular-sieving graphene oxide membranes for selective hydrogen separation. *Science* **342**, 95-98 (2013).

14. Chi, C. L. et al. Facile preparation of graphene oxide membranes for gas separation. *Chem. Mater.* **28**, 2921-2927 (2016).

15. Wang, X. R. et al. Improving the hydrogen selectivity of graphene oxide membranes by reducing non-selective pores with intergrown ZIF-8 crystals. *Chem. Commun.* **52**, 8087-8090 (2016).

16. Shen, J., Liu, G. P., Huang, K., Chu, Z. Y., Jin, W. Q. & Xu, N. P. Subnanometer two-dimensional graphene oide channels for ultrafast gas sieving. *ACS Nano* **10**, 3398-3409 (2016).

17. Guo, H. L. et al. Cross-linking between sodalite nanoparticles and graphene oxide in composite membranes to trigger high gas permeance, selectivity, and stability in hydrogen separation. *Angew. Chem. Int. Ed.* **59**, 6284-6288 (2020).

18. Huang, L., Jia, W. G. & Lin, H. Q. Etching and acidifying graphene oxide membranes to increase gas permeance while retaining molecular sieving ability. *AIChE J.* **66**, 12 (2020).

19. Achari, A., Sahana, S. & Eswaramoorth, M. High performance MoS_2 membranes: effects of thermally driven phase transition on CO_2 separation efficiency. *Energy Environ. Sci.* **9**, 1224-1228 (2016).

20. Wang, D., Wang, Z. G., Wang, L., Hu, L. & Jin, J. Ultrathin membranes of single-layered MoS_2 nanosheets for high-permeance hydrogen separation. *Nanoscale* **7**, 17649-17652 (2015).

21. Su, P. C., Tang, H. Y., Jia, M. M., Lin, Y. S. & Li, W. B. Vapor linker exchange of partially amorphous metal-organic framework membranes for ultra-selective gas separation. *AIChE J.* e17576 (2014).

22. Zhang, X. F. et al. New membrane architecture with high performance: ZIF-8 membrane supported on vertically aligned ZnO nanorods for gas permeation and separation. *Chem. Mater.* **26**, 1975-1981 (2014).

23. Bux, H., Feldhoff, A., Cravillon, J., Wiebcke, M., Li, Y. S. & Caro, J. Oriented zeolitic imidazolate framework-8 membrane with sharp H_2/C_3H_8 molecular sieve separation. *Chem. Mater.* **23**, 2262-2269 (2011).

24. Huang, K., Dong, Z. Y,. Li, Q. Q. & Jin, W. Q. Growth of a ZIF-8 membrane on the inner-surface of a ceramic hollow fiber via cycling precursors. *Chem. Commun.* **49**, 10326-10328 (2013).

25. Huang, A. S., Bux, H., Steinbach, F. & Caro, J. Molecular-sieve membrane with hydrogen permselectivity: ZIF-22 in LTA topology prepared with 3-aminopropyltriethoxysilane as covalent linker. *Angew. Chem. Int. Ed.* **49**, 4958-4961 (2010).

26. Guo, H. L., Zhu, G. S., Hewitt, I. J. & Qiu, S. L. "Twin copper source" growth of metal-organic framework membrane: $Cu_3(BTC)_2$ with high permeability and selectivity for recycling H_2. *J. Am. Chem. Soc.* **131**, 1646 (2009).

27. Zhou, S. Y. et al. Development of hydrogen-selective CAU-1 MOF membranes for

hydrogen purification by 'dual-metal-source' approach. *Int. J. Hydrogen Energy* **38**, 5338-5347 (2013).

28. Aguado, S. et al. Facile synthesis of an ultramicroporous MOF tubular membrane with selectivity towards CO_2. *New J. Chem.* **35**, 41-44 (2011).

29. Wang, N. Y,. Mundstock, A., Liu, Y., Huang, A. S. & Caro, J. Amine-modified Mg-MOF-74/CPO-27-Mg membrane with enhanced H_2/CO_2 separation. *Chem. Eng. Sci.* **124**, 27-36 (2015).

30. Knebel, A., Friebe, S., Bigall, N. C., Benzaqui, M., Serre, C. & Caro, J. Comparative study of MIL-96(Al) as continuous metal-organic frameworks layer and mixed-matrix membrane. *ACS Appl. Mater. Inter.* **8**, 7536-7544 (2016).

31. Hou, J. M., Wei, Y. Y., Zhou, S., Wang, Y. J. & Wang, H. H. Highly efficient H_2/CO_2 separation via an ultrathin metal-organic framework membrane. *Chem. Eng. Sci.* **182**, 180-188 (2018).

32. Fu, J. R., Das, S., Xing, G. L., Ben, T., Valtchev, V. & Qiu, S. L. Fabrication of COF-MOF composite membranes and their highly selective separation of H_2/CO_2. *J. Am. Chem. Soc.* **138**, 7673-7680 (2016).

33. Peng, Y. et al. Metal-organic framework nanosheets as building blocks for molecular sieving membranes. *Science* **346**, 1356-1359 (2014).

34. Li, Y. J. et al. Growth of ZnO self-converted 2D nanosheet zeolitic imidazolate framework membranes by an ammonia-assisted strategy. *Nano Res.* **11**, 1850-1860 (2018).

35. Wang, X. R. et al. Reversed thermo-switchable molecular sieving membranes composed of two-dimensional metal-organic nanosheets for gas separation. *Nat. Commun.* **8**, 10 (2017).

36. Ma, Y. N. et al. Formation of a thin and continuous MOF membrane with 2D MOF nanosheets as seeds via layer-by-layer growth. *Chem. Commun.* **55**, 10146-10149

(2019).

37. Fan, H. W., Peng, M. H., Strauss, I., Mundstock, A., Meng, H. & Caro, J. High-flux vertically aligned 2D covalent organic framework membrane with enhanced hydrogen separation. *J. Am. Chem. Soc.* **142**, 6872-6877 (2020).

38. Fan, H. W. et al. Covalent organic framework-covalent organic framework bilayer membranes for highly selective gas separation. *J. Am. Chem. Soc.* **140**, 10094-10098 (2018).

39. Das, S. & Ben, T. A COF-300-UiO-66 composite membrane with remarkably high permeability and H_2/CO_2 separation selectivity. *Dalton Trans.* **47**, 7206-7212 (2018).

40. Ding, L. et al. MXene molecular sieving membranes for highly efficient gas separation. *Nat. Commun.* **9**, 7 (2018).
